# Supplementary material for: Targeted innate immune inhibition therapy compared with antibiotics for recurrent acute cystitis: a randomized, open-label phase 2 trial
Source: Nat Microbiol. 2026 Feb 12;11(3):638–47. doi: 10.1038/s41564-026-02262-1 (PMC12962970; doi:10.1038/s41564-026-02262-1)
Supplement: Supplementary file 1 — Supplementary Figs. 1 and 2. [file 41564_2026_2262_MOESM1_ESM.pdf]

nature microbiology

Supplementary information

https://doi.org/10.1038/s41564-026-02262-1

Targeted innate immune inhibition therapy compared with antibiotics for recurrent acute cystitis: a randomized, open-label phase 2 trial

In the format provided by the authors and unedited

Supplementary Figure 1

UK English ACSS© Questionaire

First visit (diagnostic form) – Part A

Time: __ _/___ Date of evaluation: / / (dd/mm/yyyy)

		Please indicate whether you have had the following symptoms during the past 24 hours, and how severe they were: (*Please mark **only one** answer for each symptom*)	0	1	2	3
Typical Symptoms	1	Frequent urination of small volumes of urine *(going to the toilet very often)*	☐ No<br>*4 or less times per day*	☐ Yes, mild<br>*5-6 times/day*	☐ Yes, moderate<br>*7-8 times/day*	☐ Yes, severe<br>*9-10 or more times/day*
	2	Urgent urination *(a sudden and uncontrollable urge to pass urine)*	☐ No	☐ Yes, mild	☐ Yes, moderate	☐ Yes, severe
	3	Feeling pain or burning when passing urine	☐ No	☐ Yes, mild	☐ Yes, moderate	☐ Yes, severe
	4	Incomplete bladder emptying after urination	☐ No	☐ Yes, mild	☐ Yes, moderate	☐ Yes, severe
	5	Pain or uncomfortable pressure in the lower abdomen *(suprapubic area)*	☐ No	☐ Yes, mild	☐ Yes, moderate	☐ Yes, severe
	6	Visible blood in your urine	☐ No	☐ Yes, mild	☐ Yes, moderate	☐ Yes, severe
					**Sum of "Typical" scores=**	**points**
Differential	7	Loin *(low back)* pain*	☐ No	☐ Yes, mild	☐ Yes, moderate	☐ Yes, severe
	8	Vaginal discharge *(especially in the morning)*	☐ No	☐ Yes, mild	☐ Yes, moderate	☐ Yes, severe
	9	Urethral discharge *(without urination)*	☐ No	☐ Yes, mild	☐ Yes, moderate	☐ Yes, severe
	10	High body temperature *(chills/fever)*<br>*(Please indicate if measured)*	☐ No<br>≤37.5 ˚C	☐ Yes, mild<br>37.6˚-37.9 ˚C	☐ Yes, moderate<br>38.0 °C-38.9 °C	☐ Yes, severe<br>≥39.0 ˚C
		*often unilateral (on one side)			**Sum of "Differential" scores=**	**points**

Quality of life

11 **Please give an overall rating of how much these symptoms, mentioned above, bothered you in the past 24 hours *(Please mark only one answer)***

- ☐ 0 Do not feel any discomfort *(No symptoms at all. (Felt as good as usual)*
- ☐ 1 Feeling little discomfort *(Feeling somewhat worse than usual)*
- ☐ 2 Feeling moderate discomfort *(Feeling quite bad)*
- ☐ 3 Feeling extreme discomfort *(Feeling terrible)*

12 **Please choose the number, which most closely describes your normal work/everyday activities were affected by your symptoms, mentioned above, in the past 24 hours *(Please mark only one answer)***

- ☐ 0 Not affected at all *(Carrying out usual daily activities)*
- ☐ 1 Mildly affected *(Able to carry out daily activities with some discomfort)*
- ☐ 2 Moderately affected *(Only able to carry our daily activities with significant effort)*
- ☐ 3 Extremely affected *(Almost impossible to carry out daily activities)*

13 **Please indicate, how much your social activities were affected by your symptoms, mentioned above in the past 24 hours *(Please mark only one answer)***

- ☐ 0 Not affected at all *(Able to enjoy normal social activities)*
- ☐ 1 Mildly affected *(Only able to do some social activities)*
- ☐ 2 Moderately affected *(Only able to do a few social activities)*
- ☐ 3 Extremely affected *(Not able to do any social activity – symptoms keep me a "prisoner" in my home)*

Sum of "QoL" scores= points

Additional

14	Please indicate whether you have the following today		
	Menstruation *(women's monthly period)*?	☐ No	☐ Yes
	Premenstrual symptoms?	☐ No	☐ Yes
	Symptoms of the menopause*?*	☐ No	☐ Yes
	Are you pregnant?	☐ No	☐ Yes
	Do you have diabetes mellitus *(sugar diabetes)*	☐ No	☐ Yes

©The Acute Cystitis Symptom Score (ACSS) is copyrighted (www.acss.world), but free for personal use. For clinical studies or commercial usage, please ask the copyright holders (info@acss.world). Alidjanov et al 2017 JOJ uro & nephron 1(3):555561

UK English ACSS© Questionaire

Control visit (follow-up form) – Part B

Time: ___/___ Date of evaluation: / / (dd/mm/yyyy)

Please indicate if you experienced any changes in your symptoms since you last completed the first part of this questionnaire (*Please mark <u>only one</u> answer*)

Dynamics

- ☐ 0 Yes I feel normal *(All symptoms have gone away)*
- ☐ 1 Yes, I feel much better *(Most of symptoms have gone away)*
- ☐ 2 Yes, I feel somewhat better *(Only some symptoms are gone)*
- ☐ 3 No, there are barely any changes *(I still have about the same symptoms)*
- ☐ 4 Yes, I feel worse *(My condition is worse)* .

Please indicate whether you have had the following symptoms during the past 24 hours, and how severe they were:

		Please mark only one answer for each symptom	0	1	2	3
Typical Symptoms	1	Frequent urination of small volumes of urine *(going to the toilet very often)*	☐ None<br>*4 or less times per day*	☐ Yes, mild<br>*5-6 times/day*	☐ Yes, moderate<br>*7-8 times/day*	☐ Yes, severe<br>*9-10 or more times/day*
	2	Urgent urination *(a sudden and uncontrollable urge to pass urine)*	☐ None	☐ Yes, mild	☐ Yes, moderate	☐ Yes, severe
	3	Feeling pain or burning when passing urine	☐ None	☐ Yes, mild	☐ Yes, moderate	☐ Yes, severe
	4	Incomplete bladder emptying after urination	☐ None	☐ Yes, mild	☐ Yes, moderate	☐ Yes, severe
	5	Pain or uncomfortable pressure in the lower abdomen *(suprapubic area)*	☐ None	☐ Yes, mild	☐ Yes, moderate	☐ Yes, severe
	6	Visible blood in your urine	☐ None	☐ Yes, mild	☐ Yes, moderate	☐ Yes, severe
					**Sum of "Typical" scores=**	**points**
Differential	7	Loin *(low back)* pain*	☐ None	☐ Yes, mild	☐ Yes, moderate	☐ Yes, severe
	8	Vaginal discharge *(especially in the morning)*	☐ None	☐ Yes, mild	☐ Yes, moderate	☐ Yes, severe
	9	Urethral discharge (*without urination*)	☐ None	☐ Yes, mild	☐ Yes, moderate	☐ Yes, severe
	10	High high body temperature (*chills/fever*) *(Please indicate if measured)*	☐ None<br>(≤37.5 ˚C)	☐ Yes, mild<br>(37.6 ˚C-37.9 ˚C)	☐ Yes, moderate<br>(38.0 ˚C-38.9 ˚C)	☐ Yes, severe<br>(≥39,0°C)
		*often unilateral (on one side)			**Sum of "Differential" scores=**	**points**

Quality of life

11 **Please give an overall rating of how much these symptoms, mentioned above, bothered you in the past 24 hours (*Please mark <u>only one</u> answer*)**

- ☐ 0 Do not feel any discomfort *(No symptoms at all. Felt as good as usual)*
- ☐ 1 Feeling little discomfort *(Feeling somewhat worse than usual)*
- ☐ 2 Feeling moderate discomfort *(Feeling quite bad)*
- ☐ 3 Feeling extreme discomfort *(Feeling terrible)*

12 **Please choose the number, which most closely describes your normal work/everyday activities were affected by your symptoms, mentioned above, in the past 24 hours (*Please mark <u>only one</u> answer*)**

- ☐ 0 Not affected at all *(Carrying out usual daily activities)*
- ☐ 1 Mildly affected *(Able to carry out daily activities with some discomfort)*
- ☐ 2 Moderately affected *(Only able to carry out daily activities with significant effort)*
- ☐ 3 Severely affected *(Almost impossible to carry out daily activities)*

13 **Please indicate, how much your social activities were affected by your symptoms, mentioned above in the past 24 hours (*Please mark <u>only one</u> answer*)**

- ☐ 0 Not affected at all *(Able to enjoy normal social activities)*
- ☐ 1 Mildly affected *(Only able to do some social activities)*
- ☐ 2 Moderately affected *(Only able to do a few social acrivities)*
- ☐ 3 Severely affected *(Not able to do any social activities – symptoms keep me a "prisoner" in my home)*

Sum of "QoL" scores= points

Additional

14	**Please indicate whether you have the following today**		
	Menstruation *(women's monthly period)*?	☐ No	☐ Yes
	Premenstrual symptomes?	☐ No	☐ Yes
	Symptoms of the menopause ***?***	☐ No	☐ Yes
	Are you pregnant ?	☐ No	☐ Yes
	Do you have diabetes mellitus *(sugar diabetes)*	☐ No	☐ Yes

©The Acute Cystitis Symptom Score (ACSS) is copyrighted (www.acss.world), but free for personal use. For clinical studies or commercial usage, please ask the copyright holders (info@acss.world). Alidjanov et al 2017 JOJ uro & nephron 1(3):555561

Supplementary Figure 2

Cumulative Link Mixed Model fitted with the Laplace approximation
formula: response ~ treatment * time + (1 | subject)

data: Typical

link	threshold	nobs	logLik	AIC	niter	max.grad	cond.H
logit	flexible	149	-289.84	629.68	3116(11906)	7.30E-05	2.50E+03

Random effects:

Groups	Name	Variance	Std.Dev.
subject	(Intercept)	2.521	1.588
Number of groups:	subject 30		

Coefficients:

	Estimate	Std. Error	z value	Pr(>|z|)	
treatmentAnakinra	0.4035	0.9564	0.422	0.673	
timeDay15	-5.7321	1.0236	-5.600	2.14E-08	***
time6m	-5.0678	0.9915	-5.111	3.20E-07	***
timeDay5	-4.4322	0.9620	-4.607	4.08E-06	***
timeDay30	-6.9482	1.2261	-5.667	1.45E-08	***
treatmentAnakinra:timeDay15	0.5834	1.0615	0.550	0.583	
treatmentAnakinra:time6m	-0.4970	1.0603	-0.469	0.639	
treatmentAnakinra:timeDay5	0.3242	1.0343	0.313	0.754	
treatmentAnakinra:timeDay30	0.5584	1.2550	0.445	0.656	

Signif. codes: 0 '***' 0.001 '**' 0.01 '*' 0.05 '.' 0.1 ' ' 1

Threshold coefficients:

	Estimate	Std. Error	z value
0|1	-5.8937	0.9824	-6.000
1|2	-4.8433	0.9466	-5.117
2|3	-4.2354	0.9258	-4.575
3|4	-3.7804	0.9106	-4.151
4|5	-3.3451	0.8945	-3.740
5|6	-3.1381	0.8857	-3.543
6|7	-2.7620	0.8694	-3.177
7|8	-2.0057	0.8460	-2.371
8|9	-1.5645	0.8375	-1.868
9|10	-0.8608	0.8283	-1.039
10|11	0.1544	0.8232	0.188
11|12	1.2998	0.8479	1.533
12|13	1.7380	0.8693	1.999
13|14	2.2589	0.9055	2.495
14|15	2.8670	0.9656	2.969

Estimated Marginal Means

time = Day1:

contrast	estimate	SE	df	z.ratio	p.value
Nitrofurantoin - Anakinra	-0.4035	0.956	Inf	-0.422	0.6731

time = Day15:

contrast	estimate	SE	df	z.ratio	p.value
Nitrofurantoin - Anakinra	-0.9869	0.978	Inf	-1.009	0.3128

time = 6m:

contrast	estimate	SE	df	z.ratio	p.value
Nitrofurantoin - Anakinra	0.0934	0.975	Inf	0.096	0.9237

time = Day5:

contrast	estimate	SE	df	z.ratio	p.value
Nitrofurantoin - Anakinra	-0.7277	0.941	Inf	-0.773	0.4393

time = Day30:

contrast	estimate	SE	df	z.ratio	p.value
Nitrofurantoin - Anakinra	-0.9619	1.180	Inf	-0.815	0.4153

treatment = Nitrofurantoin:

contrast	estimate	SE	df	z.ratio	p.value
timeDay1 - timeDay15	5.732	1.020	Inf	5.600	<.0001
timeDay1 - time6m	5.068	0.992	Inf	5.111	<.0001
timeDay1 - timeDay5	4.432	0.962	Inf	4.607	<.0001
timeDay1 - timeDay30	6.948	1.230	Inf	5.667	<.0001
timeDay15 - time6m	-0.664	0.887	Inf	-0.749	0.9448
timeDay15 - timeDay5	-1.300	0.863	Inf	-1.505	0.5589
timeDay15 - timeDay30	1.216	1.070	Inf	1.133	0.7890
time6m - timeDay5	-0.636	0.865	Inf	-0.735	0.9484
time6m - timeDay30	1.880	1.090	Inf	1.718	0.4230
timeDay5 - timeDay30	2.516	1.070	Inf	2.343	0.1314

treatment = Anakinra:

contrast	estimate	SE	df	z.ratio	p.value
timeDay1 - timeDay15	5.149	0.752	Inf	6.850	<.0001
timeDay1 - time6m	5.565	0.775	Inf	7.181	<.0001
timeDay1 - timeDay5	4.108	0.695	Inf	5.907	<.0001
timeDay1 - timeDay30	6.390	0.836	Inf	7.645	<.0001
timeDay15 - time6m	0.416	0.592	Inf	0.703	0.9559
timeDay15 - timeDay5	-1.041	0.578	Inf	-1.799	0.3739
timeDay15 - timeDay30	1.241	0.639	Inf	1.941	0.2953
time6m - timeDay5	-1.457	0.575	Inf	-2.533	0.0833
time6m - timeDay30	0.825	0.621	Inf	1.328	0.6739
timeDay5 - timeDay30	2.282	0.632	Inf	3.611	0.0028

P value adjustment: tukey method for comparing a family of 5 estimates

Cumulative Link Mixed Model fitted with the Laplace approximation
formula: response ~ treatment * time + (1 | subject)

data: QoL

link	threshold	nobs	logLik	AIC	niter	max.grad	cond.H
logit	flexible	149	-224.46	486.91	1688(6533)	3.71E-06	1.00E+03

Random effects:

Groups	Name	Variance	Std.Dev.
subject	(Intercept)	2.531	1.591
Number of groups:	subject 30		

Coefficients:

	Estimate	Std. Error	z value	Pr(>|z|)	
treatmentAnakinra	0.04429	0.95432	0.046	0.962982	
timeDay15	-5.07504	1.0894	-4.659	3.18E-06	***
time6m	-3.73666	0.99629	-3.751	0.000176	***
timeDay5	-3.15295	0.91065	-3.462	0.000536	***
timeDay30	-5.45464	1.19751	-4.555	5.24E-06	***
treatmentAnakinra:timeDay15	0.67341	1.16097	0.580	0.561886	
treatmentAnakinra:time6m	-0.48119	1.11706	-0.431	0.666637	
treatmentAnakinra:timeDay5	-0.28173	1.01912	-0.276	0.782210	
treatmentAnakinra:timeDay30	0.07455	1.29773	0.057	0.954191	

Signif. codes: 0 '***' 0.001 '**' 0.01 '*' 0.05 '.' 0.1 ' ' 1

Threshold coefficients:

	Estimate	Std. Error	z value
0|1	-4.194	0.9279	-4.520
1|2	-3.2477	0.8962	-3.624
2|3	-2.8444	0.8822	-3.224
3|4	-1.6828	0.8416	-2.000
4|5	-0.9281	0.8228	-1.128
5|6	-0.4771	0.8171	-0.584
6|7	0.6618	0.8236	0.804
7|8	2.1401	0.9002	2.377
8|9	2.6736	0.9547	2.800

Estimated Marginal Means

time = Day1:

contrast	estimate	SE	df	z.ratio	p.value
Nitrofurantoin - Anakinra	-0.0443	0.954	Inf	-0.046	0.9630

time = Day15:

contrast	estimate	SE	df	z.ratio	p.value
Nitrofurantoin - Anakinra	-0.7177	1.080	Inf	-0.662	0.5082

time = 6m:

contrast	estimate	SE	df	z.ratio	p.value
Nitrofurantoin - Anakinra	0.4369	1.050	Inf	0.417	0.6770

time = Day5:

contrast	estimate	SE	df	z.ratio	p.value
Nitrofurantoin - Anakinra	0.2374	0.931	Inf	0.255	0.7987

time = Day30:

contrast	estimate	SE	df	z.ratio	p.value

Nitrofurantoin - Anakinra	-0.1188	1.230	Inf	-0.097	0.9229
treatment = Nitrofurantoin:					
**contrast**	**estimate**	**SE**	**df**	**z.ratio**	**p.value**
timeDay1 - timeDay15	5.075	1.090	Inf	4.659	<.0001
timeDay1 - time6m	3.737	0.996	Inf	3.751	0.0017
timeDay1 - timeDay5	3.153	0.911	Inf	3.462	0.0049
timeDay1 - timeDay30	5.455	1.200	Inf	4.555	0.0001
timeDay15 - time6m	-1.338	1.040	Inf	-1.289	0.6981
timeDay15 - timeDay5	-1.922	0.951	Inf	-2.022	0.2554
timeDay15 - timeDay30	0.380	1.160	Inf	0.327	0.9975
time6m - timeDay5	-0.584	0.900	Inf	-0.648	0.9670
time6m - timeDay30	1.718	1.140	Inf	1.502	0.5609
timeDay5 - timeDay30	2.302	1.060	Inf	2.171	0.1907
treatment = Anakinra:					
**contrast**	**estimate**	**SE**	**df**	**z.ratio**	**p.value**
timeDay1 - timeDay15	4.402	0.729	Inf	6.041	<.0001
timeDay1 - time6m	4.218	0.734	Inf	5.749	<.0001
timeDay1 - timeDay5	3.435	0.671	Inf	5.117	<.0001
timeDay1 - timeDay30	5.380	0.839	Inf	6.413	<.0001
timeDay15 - time6m	-0.184	0.646	Inf	-0.284	0.9986
timeDay15 - timeDay5	-0.967	0.607	Inf	-1.594	0.5012
timeDay15 - timeDay30	0.978	0.731	Inf	1.338	0.6671
time6m - timeDay5	-0.783	0.614	Inf	-1.275	0.7066
time6m - timeDay30	1.162	0.736	Inf	1.579	0.5112
timeDay5 - timeDay30	1.945	0.708	Inf	2.747	0.0475

P value adjustment: tukey method for comparing a family of 5 estimates

Cumulative Link Mixed Model fitted with the Laplace approximation
formula: response ~ treatment * time + (1 | subject)

data: Differential

link	threshold	nobs	logLik	AIC	niter	max.grad	cond.H
logit	flexible	149	-121.18	272.37	978(4662)	8.93E-06	3.10E+02

Random effects:

Groups	Name	Variance	Std.Dev.
subject	(Intercept)	1.682	1.297
Number of groups:	subject 30		

Coefficients:

	Estimate	Std. Error	z value	Pr(>|z|)	
treatmentAnakinra	-1.7896	0.9439	-1.896	0.057972	.
timeDay15	-4.3274	1.2603	-3.434	0.000596	***
time6m	-3.5586	1.1287	-3.153	0.001617	**
timeDay5	-2.9263	1.0133	-2.888	0.003876	**
timeDay30	-3.9777	1.1877	-3.349	0.000811	***
treatmentAnakinra:timeDay15	1.5332	1.4994	1.023	0.306525	
treatmentAnakinra:time6m	2.3197	1.2901	1.798	0.072167	.
treatmentAnakinra:timeDay5	0.6028	1.2671	0.476	0.634250	
treatmentAnakinra:timeDay30	1.2129	1.4543	0.834	0.404248	

Signif. codes: 0 ‘***’ 0.001 ‘**’ 0.01 ‘*’ 0.05 ‘.’ 0.1 ‘ ’ 1

Threshold coefficients:

	Estimate	Std. Error	z value
0|1	-1.9696	0.7883	-2.498
1|2	-0.6364	0.7502	-0.848
2|3	0.3125	0.7655	0.408
3|4	1.6307	0.8551	1.907
4|5	2.5800	1.0014	2.577
5|6	-1.9696	0.7883	-2.498

Estimated Marginal Means

time = Day1:

contrast	estimate	SE	df	z.ratio	p.value
Nitrofurantoin - Anakinra	1.790	0.944	Inf	1.896	0.0580

time = Day15:

contrast	estimate	SE	df	z.ratio	p.value
Nitrofurantoin - Anakinra	0.256	1.330	Inf	0.193	0.8472

time = 6m:

contrast	estimate	SE	df	z.ratio	p.value
Nitrofurantoin - Anakinra	-0.530	1.120	Inf	-0.475	0.6350

time = Day5:

contrast	estimate	SE	df	z.ratio	p.value
Nitrofurantoin - Anakinra	1.187	1.11	Inf	1.066	0.2863

time = Day30:

contrast	estimate	SE	df	z.ratio	p.value
Nitrofurantoin - Anakinra	0.577	1.300	Inf	0.444	0.6574

treatment = Nitrofurantoin:

contrast	estimate	SE	df	z.ratio	p.value
timeDay1 - timeDay15	4.3274	1.260	Inf	3.434	0.0054

timeDay1 - time6m	3.5586	1.130	Inf	3.153	0.0140
timeDay1 - timeDay5	2.9263	1.010	Inf	2.888	0.0317
timeDay1 - timeDay30	3.9777	1.190	Inf	3.349	0.0072
timeDay15 - time6m	-0.7688	1.250	Inf	-0.615	0.9727
timeDay15 - timeDay5	-1.4010	1.170	Inf	-1.193	0.7556
timeDay15 - timeDay30	-0.3497	1.240	Inf	-0.282	0.9986
time6m - timeDay5	-0.6323	1.110	Inf	-0.572	0.9792
time6m - timeDay30	0.4191	1.210	Inf	0.347	0.9969
timeDay5 - timeDay30	1.0514	1.120	Inf	0.936	0.8828
treatment = Anakinra:					
**contrast**	**estimate**	**SE**	**df**	**z.ratio**	**p.value**
timeDay1 - timeDay15	2.7942	0.934	Inf	2.991	0.0233
timeDay1 - time6m	1.2389	0.693	Inf	1.787	0.3813
timeDay1 - timeDay5	2.3235	0.835	Inf	2.782	0.0430
timeDay1 - timeDay30	2.7648	0.954	Inf	2.898	0.0308
timeDay15 - time6m	-1.5553	0.927	Inf	-1.679	0.4472
timeDay15 - timeDay5	-0.4707	1.020	Inf	-0.461	0.9908
timeDay15 - timeDay30	-0.0295	1.120	Inf	-0.026	1.0000
time6m - timeDay5	1.0846	0.829	Inf	1.309	0.6856
time6m - timeDay30	1.5258	0.947	Inf	1.611	0.4905
timeDay5 - timeDay30	0.4412	1.040	Inf	0.425	0.9932

P value adjustment: tukey method for comparing a family of 5 estimates

Analysis of Variance of Aligned Rank Transformed Data

Table Type: Analysis of Deviance Table (Type III Wald F tests with Kenward-Roger df)
Model: Mixed Effects (lmer)
Response: art(response)

data: Leukocytes

		F	Df	Df.res	Pr(>F)	
1	treatment	1.2977	1	27.951	0.2642950	
2	time	4.6581	4	107.152	0.0016628	**
3	treatment:time	1.4002	4	107.155	0.2388621	

Signif. codes: 0 '***' 0.001 '**' 0.01 '*' 0.05 '.' 0.1 ' ' 1

data: Neutrophils

		F	Df	Df.res	Pr(>F)	
1	treatment	1.1385	1	27.772	0.29516723	
2	time	5.2589	4	97.981	0.00070289	***
3	treatment:time	1.1352	4	98.004	0.34449468	

Signif. codes: 0 '***' 0.001 '**' 0.01 '*' 0.05 '.' 0.1 ' ' 1

data: Lymphocytes

		F	Df	Df.res	Pr(>F)	
1	treatment	1.6172	1	27.865	0.213990	
2	time	1.4348	4	97.529	0.228248	
3	treatment:time	2.1269	4	97.591	0.083182	.

Signif. codes: 0 '***' 0.001 '**' 0.01 '*' 0.05 '.' 0.1 ' ' 1
