# Supplementary material for: Targeted innate immune inhibition therapy compared with antibiotics for recurrent acute cystitis: a randomized, open-label phase 2 trial
Source: Nat Microbiol. 2026 Feb 12;11(3):638–47. doi: 10.1038/s41564-026-02262-1 (PMC12962970; doi:10.1038/s41564-026-02262-1)
Supplement: Supplementary file 2 — Reporting Summary [file 41564_2026_2262_MOESM2_ESM.pdf]

nature portfolio

Corresponding author(s): Ines Ambite

Last updated by author(s): Jan 8, 2026

Reporting Summary

Nature Portfolio wishes to improve the reproducibility of the work that we publish. This form provides structure for consistency and transparency in reporting. For further information on Nature Portfolio policies, see our Editorial Policies and the Editorial Policy Checklist.

Statistics

For all statistical analyses, confirm that the following items are present in the figure legend, table legend, main text, or Methods section.

n/a	Confirmed	
☐	☒	The exact sample size (*n*) for each experimental group/condition, given as a discrete number and unit of measurement
☐	☒	A statement on whether measurements were taken from distinct samples or whether the same sample was measured repeatedly
☐	☒	The statistical test(s) used AND whether they are one- or two-sided<br>*Only common tests should be described solely by name; describe more complex techniques in the Methods section.*
☒	☐	A description of all covariates tested
☐	☒	A description of any assumptions or corrections, such as tests of normality and adjustment for multiple comparisons
☐	☒	A full description of the statistical parameters including central tendency (e.g. means) or other basic estimates (e.g. regression coefficient) AND variation (e.g. standard deviation) or associated estimates of uncertainty (e.g. confidence intervals)
☐	☒	For null hypothesis testing, the test statistic (e.g. *F*, *t*, *r*) with confidence intervals, effect sizes, degrees of freedom and *P* value noted<br>*Give P values as exact values whenever suitable.*
☒	☐	For Bayesian analysis, information on the choice of priors and Markov chain Monte Carlo settings
☒	☐	For hierarchical and complex designs, identification of the appropriate level for tests and full reporting of outcomes
☒	☐	Estimates of effect sizes (e.g. Cohen's *d*, Pearson's *r*), indicating how they were calculated

Our web collection on statistics for biologists contains articles on many of the points above.

Software and code

Policy information about availability of computer code

Data collection	Gene expression microarray - GeneTitan System (ThermoFisher Scientific)
Data analysis	Statistics - Code used for statistical analysis is available at CodeOcean (doi.org/10.24433/CO.8500789.v1); Prism version 10.5.0 for macOS (GraphPad Software); R version 4.5.1; R Studio version 2025.05.01 Build 513 (Posit Software, PBC); Microsoft Excel for Mac version 16.96<br>Transcriptomic data - Transcriptome Analysis console (TAC) version 4.0.1.36. (Applied Biosystems); Ingenuity Pathway Analysis Version 01-23-01 (Qiagen)

For manuscripts utilizing custom algorithms or software that are central to the research but not yet described in published literature, software must be made available to editors and reviewers. We strongly encourage code deposition in a community repository (e.g. GitHub). See the Nature Portfolio guidelines for submitting code & software for further information.

Data

Policy information about availability of data

All manuscripts must include a data availability statement. This statement should provide the following information, where applicable:

- Accession codes, unique identifiers, or web links for publicly available datasets
- A description of any restrictions on data availability
- For clinical datasets or third party data, please ensure that the statement adheres to our policy

Gene expression data generated in this study are available at NCBI Gene Expression Omnibus (GEO) repository GSE315861. All clinical data supporting the findings

of this study and the study protocol are available in the Article and Supplementary Information. Source data are provided with this paper. All other data that support the findings of this study are available from the corresponding author upon reasonable request. De-identified individual and/or study-level data will be shared with researchers who provide a methodologically sound proposal and if regulatory criteria are met. Access to anonymized data may be granted following review (time frame <20 office days) to ensure compliance with relevant ethical and legal considerations.

Research involving human participants, their data, or biological material

Policy information about studies with human participants or human data. See also policy information about sex, gender (identity/presentation), and sexual orientation and race, ethnicity and racism.

Reporting on sex and gender	Rationale for female patients<br>The disease uncomplicated (recurrent) cystitis occurs only in women. Therefore, only female patients were included in this clinical trial.
Reporting on race, ethnicity, or other socially relevant groupings	Ethnicity information was self-reported by the study participants and recorded in the CRF.
Population characteristics	Demographic data, medical history, current diagnosis, physical examination and health parameters were recorded by the study physicians at enrollment in the CRF and closely monitored by an external monitor. No significant differences between the two study arms was detected.
Recruitment	Participants were recruited at the Clinic for Urology, Paediatric Urology and Andrology in Giessen, Germany, using mailing list of patients from the bladder consultation clinic and advertisement on the study website. All potentially suitable patients were screened. To avoid self biases, potential participants were recruited consecutively from those who presented at screening. Inclusion and exclusion criteria were carefully defined in the study protocol. Written information was presented to the patients, and participation required a signed informed consent.
Ethics oversight	Ethical approval was obtained from the German Ethical Review Authority (ethics vote AZ10/21). The trial was conducted in accordance with the principles of the Declaration of Helsinki principles, the International Council for Harmonisation guidelines for good clinical practice, and applicable national laws and regulatory requirements.

Note that full information on the approval of the study protocol must also be provided in the manuscript.

Field-specific reporting

Please select the one below that is the best fit for your research. If you are not sure, read the appropriate sections before making your selection.

☒ Life sciences ☐ Behavioural & social sciences ☐ Ecological, evolutionary & environmental sciences

For a reference copy of the document with all sections, see nature.com/documents/nr-reporting-summary-flat.pdf

Life sciences study design

All studies must disclose on these points even when the disclosure is negative.

Sample size	The primary objective of the trial is to evaluate the safety and efficacy of Anakinra treatment for episodes of acute cystitis in patients with recurrent disease. No formal sample size calculation evaluating the power of the trial has been performed. However, a consideration regarding the sample size was made based on previous studies of Anakinra in a murine acute cystitis model.<br>For efficacy, the sample size was based on analysis of symptom scores, inflammatory parameters and bacterial cultures. A sample size of 20 patients in the Anakinra treatment group and ten in the group receiving antibiotics was deemed suitable to achieve criterion for significance (alpha) 0.05 and power 90% using the paired samples 1-tailed t-test. The null hypothesis is H0: mean change in symptom score = 0 and the alternative hypothesis is HA: mean change in symptom score > 0.
Data exclusions	No data was excluded.
Replication	Replication of clinical study was not relevant.
Randomization	All enrolled subjects were randomized to two study treatment groups at Visit 1, the chance for allocation to the anakinra group or nitrofurantoin group was 2:1. Randomization was performed by the central office of the Center for Clinical Trials of the Philipps-University Marburg.
Blinding	The administrated Investigational product was then documented together with date, time and study code in the source documentation and eCRF. The analysis of the study parameters was blinded.

Reporting for specific materials, systems and methods

We require information from authors about some types of materials, experimental systems and methods used in many studies. Here, indicate whether each material, system or method listed is relevant to your study. If you are not sure if a list item applies to your research, read the appropriate section before selecting a response.

Materials & experimental systems

n/a	Involved in the study
☒	☐ Antibodies
☒	☐ Eukaryotic cell lines
☒	☐ Palaeontology and archaeology
☒	☐ Animals and other organisms
☐	☒ Clinical data
☒	☐ Dual use research of concern
☒	☐ Plants

Methods

n/a	Involved in the study
☒	☐ ChIP-seq
☒	☐ Flow cytometry
☒	☐ MRI-based neuroimaging

Clinical data

Policy information about clinical studies

All manuscripts should comply with the ICMJE guidelines for publication of clinical research and a completed CONSORT checklist must be included with all submissions.

Clinical trial registration: German Clinical Trials Register, DRKS00025964; EudraCT, 2019-004209-28

Study protocol: The trial protocol and statistical analysis plan can be shared upon academic or research request.

Data collection: For this randomized, open label, single-center, two-arm, parallel group, Phase II trial, patients were enrolled at the Clinic for Urology, Paediatric Urology and Andrology in Giessen, Germany. The trial was registered on July 27, 2021 at the German Clinical Trials Register #DRKS00025964 (https://drks.de/search/en/trial/DRKS00025964) and the study was conducted between September 2, 2021 and September 3, 2024. Diagnosis and treatment followed an established clinical pathway and mandated routine tests included urinalysis, full blood count and urine and blood sampling for molecular analyses.

Outcomes: The primary objective of the trial is to evaluate the safety and efficacy of Anakinra treatment for episodes of acute cystitis in patients with recurrent disease.
Primary Endpoint was the reduction in symptom score measured by the Acute Cystitis Symptom Score (ACSS) in the first 5 days.
Secondary Endpoints were recurrent UTI episodes until 6 months, bacteriuria and leucocyturia at visits until 26 weeks.

Plants

Seed stocks: *Report on the source of all seed stocks or other plant material used. If applicable, state the seed stock centre and catalogue number. If plant specimens were collected from the field, describe the collection location, date and sampling procedures.*

Novel plant genotypes: *Describe the methods by which all novel plant genotypes were produced. This includes those generated by transgenic approaches, gene editing, chemical/radiation-based mutagenesis and hybridization. For transgenic lines, describe the transformation method, the number of independent lines analyzed and the generation upon which experiments were performed. For gene-edited lines, describe the editor used, the endogenous sequence targeted for editing, the targeting guide RNA sequence (if applicable) and how the editor was applied.*

Authentication: *Describe any authentication procedures for each seed stock used or novel genotype generated. Describe any experiments used to assess the effect of a mutation and, where applicable, how potential secondary effects (e.g. second site T-DNA insertions, mosiacism, off-target gene editing) were examined.*
