# Supplementary material for: Targeted innate immune inhibition therapy compared with antibiotics for recurrent acute cystitis: a randomized, open-label phase 2 trial
Source: Nat Microbiol. 2026 Feb 12;11(3):638–47. doi: 10.1038/s41564-026-02262-1 (PMC12962970; doi:10.1038/s41564-026-02262-1)
Supplement: Supplementary file 4 — Study protocol. [file 41564_2026_2262_MOESM4_ESM.pdf]

Study Protocol

Immunomodulatory treatment of recurrent cystitis

An open label, randomized, single-center, phase II trial

Authors: Catharina Svanborg, MD, Professor (SelectImmune Pharma AB), Florian Wagenlehner, MD, Professor (Justus Liebig University Gießen), Helena Lomberg (SelectImmune Pharma AB), Gabriela Godaly (SelectImmune Pharma AB)

**EudraCT:**	2019-004209-28
**Protocol Code:**	SI002-001
**Registration**	DRKS
**Investigational active ingredients:**	Kineret®<br>Nitrofurantoin Uro-Tablinen®
**Sponsor:**	**SelectImmune Pharma AB, Lund (Sweden)**<br>Org nr: 559104-6874<br>BMC D10<br>Klinikgatan 32<br>222 42 Lund<br>Sweden
**Contractor:**	**Koordinierungszentrum für Klinische Studien (KKS)**<br>Justus-Liebig-University Giessen<br>Klinikstr. 29<br>35392Giessen
**Coordinating Investigator:**	Prof. Dr. F. Wagenlehner<br>Justus-Liebig-University Giessen<br>Clinic and Polyclinic for Urology, Pediatric Urology and Andrology

THIS DOCUMENT CONTAINS TRADE SECRETS, AND/OR COMMERCIAL INFORMATION, DELIVERED IN CONFIDENCE AND RELIANCE THAT SUCH INFORMATION WILL NOT BE COPIED OR MADE AVAILABLE TO ANY THIRD PARTY WITHOUT THE WRITTEN CONSENT OF SelectImmune PHARMA AB.

Rudolf-Buchheim-Strasse 7
35392 Giessen, Germany

Version: V07F

Date: 30.01.2023

This Clinical Trial Protocol (CTP) contains information that is confidential and the exclusive property of Prof. C. Svanborg, Prof. F. Wagenlehner and SelectImmune Pharma AB. This information is being provided to the recipient for the purpose of conducting the trial for SelectImmune Pharma AB.The content of the CTP may not be disclosed to a third party without prior written permission of SelectImmune Pharma AB and the Coordinating Investigator.

THIS DOCUMENT CONTAINS TRADE SECRETS, AND/OR COMMERCIAL INFORMATION, DELIVERED IN CONFIDENCE AND RELIANCE THAT SUCH INFORMATION WILL NOT BE COPIED OR MADE AVAILABLE TO ANY THIRD PARTY WITHOUT THE WRITTEN CONSENT OF SelectImmune PHARMA AB.

Consent Statement to Study Protocol

I have read this protocol and agree to conduct the study in accordance with all commitments of this protocol as well as with the current version of the Declaration of Helsinki, ICH-GCP E6 R2 Guideline (International Conference on Harmonization - Good Clinical Practice) and applicable national laws and regulatory requirements. I also agree to handle all information concerning this study confidentially.

Principal Investigator:

15.02.2023

Date

Prof. Florian Wagenlehner, MD, PhD

Site and Printed Name

THIS DOCUMENT CONTAINS TRADE SECRETS, AND/OR COMMERCIAL INFORMATION, DELIVERED IN CONFIDENCE AND RELIANCE THAT SUCH INFORMATION WILL NOT BE COPIED OR MADE AVAILABLE TO ANY THIRD PARTY WITHOUT THE WRITTEN CONSENT OF SelectImmune PHARMA AB.

Protocol Approval

The Sponsor

SelectImmune Pharma AB

20230310
Date

Prof. Catharina Svanborg, MD, PhD
Medical Responsible / SelectImmune Pharma AB

THIS DOCUMENT CONTAINS TRADE SECRETS, AND/OR COMMERCIAL INFORMATION, DELIVERED IN CONFIDENCE AND RELIANCE THAT SUCH INFORMATION WILL NOT BE COPIED OR MADE AVAILABLE TO ANY THIRD PARTY WITHOUT THE WRITTEN CONSENT OF SelectImmune PHARMA AB.

List of Contacts

**Coordinating Investigator**<br><br>**(LKP according to §40 AMG**)	Dr Florian Wagenlehner, MD, Professor<br>Justus-Liebig-University Giessen<br><br>Clinic and Polyclinic for Urology, Pediatric Urology and Andrology<br>Rudolf-Buchheim-Strasse 7<br>35392 Giessen, Germany
**Sponsor**<br><br><br><br><br><br><br>Sponsor Medical Expert<br><br><br>Contractor	**SelectImmune Pharma AB, Lund (Sweden)**<br>Org nr: 559104-6874<br>BMC D10<br>Klinikgatan 32<br>222 42 Lund<br>Sweden<br><br><br>Prof Catharina Svanborg, MD, PhD<br><br><br>Coordinating Center for Clinical Trials of the Philipps-University of Marburg with a branch office at the Justus-Liebig-University Giessen<br>Klinikstrasse 29<br>35392 Giessen, Germany
**Project Management**<br><br>**Including on-site monitoring**	Coordinating Center for Clinical Trials of the Philipps-University of Marburg with a branch office at the Justus-Liebig-University Giessen<br>Klinikstrasse 29<br>35392 Giessen, Germany
**Medical Monitor**	PD Dr. med. Adrian Pilatz<br>Justus Liebig University Giessen<br>Clinic and Polyclinic for Urology, Pediatric Urology and Andrology<br>Rudolf-Buchheim-Strasse 7<br>35392 Giessen, Germany
**SAE-Management /**<br><br>**Contact for SAE reporting**	**KKS Marburg**<br><br>Safety Division<br>Karl-von-Frisch-Straße 4<br>35043 Marburg<br>Phone: +49 (0)6421-28 66554 or -28 26525<br>Fax: +49 (0)6421-28 66559<br>E-mail: sae-management@kks.uni-marburg.de

THIS DOCUMENT CONTAINS TRADE SECRETS, AND/OR COMMERCIAL INFORMATION, DELIVERED IN CONFIDENCE AND RELIANCE THAT SUCH INFORMATION WILL NOT BE COPIED OR MADE AVAILABLE TO ANY THIRD PARTY WITHOUT THE WRITTEN CONSENT OF SelectImmune PHARMA AB.

**Data Management**	**KKS Marburg**<br>Data Management<br>Karl-von-Frisch-Straße 4<br>35043 Marburg<br>support@kks.uni-marburg.de
**Randomization**	KKS Marburg<br>Karl-von-Frisch-Straße 4<br>D-35043 Marburg<br>rando@kks.uni-marburg.de
**Central Lab**	Central Clinical Chemistry Laboratory, Giessen

THIS DOCUMENT CONTAINS TRADE SECRETS, AND/OR COMMERCIAL INFORMATION, DELIVERED IN CONFIDENCE AND RELIANCE THAT SUCH INFORMATION WILL NOT BE COPIED OR MADE AVAILABLE TO ANY THIRD PARTY WITHOUT THE WRITTEN CONSENT OF SelectImmune PHARMA AB.

Table of Contents

Table of Contents 7
1 Trial Identification 18
2 Introduction and Rationale 18
2.1 Background 18
2.2 Clinical experience with Kineret 19
2.3 Pre-clinical findings 20
2.4 Trial Rationale 20
2.4.1 Overall Trial Rationale 20
2.4.2 Rationale for safety 21
2.4.3 Rationale for efficacy 21
2.4.4 Rationale for female patients 21
2.4.5 Rationale for lifestyle considerations 22
2.5 Ethical and Legal Consideration Statement 22
2.5.1 Potential Risks and Benefits 22
3 Trial Objectives and Endpoints 23
3.1 Objectives 24
3.1.1 Primary Efficacy Objective 24
3.1.2 Secondary Efficacy Objectives 24
3.1.3 Safety Objective 24
3.2 Endpoints 24
3.2.1 Primary Efficacy Endpoint 24
3.2.2 Safety Endpoints 24
3.2.3 Secondary Endpoints 24
3.2.4 Exploratory Efficacy Endpoints 25
4 Trial Design 25
4.1 Overall Trial Design 25
4.2 Number of Trial Subjects 25
4.3 Subject identification and randomisation of treatment 25
4.3.1 Subject identification 25
4.3.2 Labelling, randomisation and treatment allocation to the study subject 26
5 Trial Population and Withdrawal 27
5.1 Subject Eligibility 27
5.2 Inclusion Criteria for All Subjects 27
5.3 Exclusion Criteria for All Subjects 28

THIS DOCUMENT CONTAINS TRADE SECRETS, AND/OR COMMERCIAL INFORMATION, DELIVERED IN CONFIDENCE AND RELIANCE THAT SUCH INFORMATION WILL NOT BE COPIED OR MADE AVAILABLE TO ANY THIRD PARTY WITHOUT THE WRITTEN CONSENT OF SelectImmune PHARMA AB.

5.4 Subject Enrolment Log 29
5.5 Subject Identification List 29
5.6 Discontinuation Criteria 29
5.6.1 Withdrawal of patients from study treatment 29
5.6.2 Premature discontinuation of the study 30
6 Trial Schedule and Assessments 31
6.1 Schedule of Trial Procedures 31
6.2 Description of Trial Visits 35
6.3 Demographics 39
6.4 Vital Signs 39
6.5 Physical Examination 40
6.6 Medical History and Concurrent Medical Conditions 40
6.7 Concomitant Medication 40
6.8 Pregnancy Test 41
6.9 Adverse Events 41
6.10 Laboratory Assessments 41
6.10.1 Safety Blood Tests (hematology, biochemistry) 41
6.10.2 Safety Urine analysis 42
6.10.3 Urine Samples for efficacy 43
6.10.4 Additional samples for laboratory analysis 43
6.10.5 Clinical procedures in pandemics (COVID-19) 43
7 Adverse Events 44
7.1 Documentation of Adverse Events 44
7.2 Other Events to be Reported 45
7.2.1 Pregnancy 45
7.3 Documentation and reporting of Serious Adverse Events (SAEs) 45
7.4 End date of a Serious Adverse Event 47
7.5 Exceptions from SAE Reporting 47
7.6 SUSAR Reporting Procedure 47
7.7 Sponsor Reporting Responsibilities 47
8 Investigational Product(s) 48
8.1 Investigational Medicinal Product (IMP) – Kineret® 48
8.2 Manufacturing, packaging and labelling of Kineret® 49
8.2.1 Selection of dose 50

THIS DOCUMENT CONTAINS TRADE SECRETS, AND/OR COMMERCIAL INFORMATION, DELIVERED IN CONFIDENCE AND RELIANCE THAT SUCH INFORMATION WILL NOT BE COPIED OR MADE AVAILABLE TO ANY THIRD PARTY WITHOUT THE WRITTEN CONSENT OF SelectImmune PHARMA AB.

8.2.2 Potential Toxicity in Patients ... 50
8.2.3 Manufacturer of study medication ... 51
8.2.4 Drug Supply ... 51
8.2.5 Handling and storage of the medication ... 52
8.2.6 Application of the study medicine ... 52
8.3 Administration of Investigational Product Kineret®: ... 53
8.3.1 Dose and adjustment of study treatment ... 53
8.3.2 Treatment after termination of study ... 53
8.3.3 Management of toxicities of IMP ... 53
8.3.4 Reference Document- Kineret® ... 54
8.3.5 Allowed concomitant medication ... 54
8.4 Investigational Medicinal Product (IMP) - Nitrofurantoin Uro-Tablinen® ... 54
8.4.1 Selection of dose ... 55
8.4.2 Potential Toxicity in Patients ... 55
8.4.3 Authorization holder of study medication ... 58
8.4.4 Drug Supply ... 58
8.4.5 Handling and storage of the medication ... 58
8.4.6 Application of the study medicine ... 58
8.4.7 Dose and adjustment of study treatment ... 59
8.4.8 Treatment after termination of study ... 59
8.4.9 Management of toxicities of IMP ... 59
8.4.10 Reference Document- Nitrofurantoin Uro-Tablinen® ... 59
8.4.11 Allowed concomitant medication ... 59
8.5 Drug Accountability and Compliance Checks ... 60
8.5.1 Drug Accountability Investigational Product ... 60
8.5.2 Trial Product Destruction ... 60
9 Statistical Methods ... 60
9.1 Determination of Sample Size ... 60
9.2 Definition of Trial Analysis Sets ... 61
9.3 Statistical Analysis ... 61
9.3.1 Clinical Laboratory Evaluation ... 61
9.3.2 General Principles ... 62
10 Data management ... 62
10.1 Data Protection ... 62
10.2 EDC-System (e-CRF) ... 62
11 Administration ... 64

THIS DOCUMENT CONTAINS TRADE SECRETS, AND/OR COMMERCIAL INFORMATION, DELIVERED IN CONFIDENCE AND RELIANCE THAT SUCH INFORMATION WILL NOT BE COPIED OR MADE AVAILABLE TO ANY THIRD PARTY WITHOUT THE WRITTEN CONSENT OF SelectImmune PHARMA AB.

11.1 Source data and patient files 64
11.2 Investigator Site File (ISF) and archiving 65
11.3 Monitoring, audit and inspection 66
11.3.1 Monitoring 66
11.3.2 Audit / Inspections 67
11.4 Protocol violation and discrepancies 67
12 Handling of an Urgent Safety Measure 67
13 Completion of Trial 68
13.1 Premature Termination of the Trial and/or Trial Site 68
13.2 Provision for Subject Care Following Trial Completion 68
14 Ethical considerations 68
15 Ethical and regulatory aspects 68
15.1 Ethics Committee (EC) 68
15.2 Competent Authorities (CA) 69
15.2.1 Application to the national competent authority 69
15.2.2 Notification to the local competent authority 69
15.3 Pre-conditions before study start 69
15.4 Patient insurance 70
15.5 Investigator’s responsibilities 71
15.6 Further responsibilities 72
15.7 Patient information and consent 72
15.8 Changes to the study protocol 73
15.9 Safety of subjects, immediate danger 74
16 Financial aspects 74
17 Final report 74
18 Registration 75
19 Publication 75
20 Use of Information 75
21 References 76
22 List of Appendices 77
22.1 Appendix 1: Definitions and Terminology Associated with Clinical Safety Reporting in Clinical Trials 78

THIS DOCUMENT CONTAINS TRADE SECRETS, AND/OR COMMERCIAL INFORMATION, DELIVERED IN CONFIDENCE AND RELIANCE THAT SUCH INFORMATION WILL NOT BE COPIED OR MADE AVAILABLE TO ANY THIRD PARTY WITHOUT THE WRITTEN CONSENT OF SelectImmune PHARMA AB.

22.2 Appendix 2: Classification of Adverse Events 80
22.3 ACSS Questionnaire (German) 83

THIS DOCUMENT CONTAINS TRADE SECRETS, AND/OR COMMERCIAL INFORMATION, DELIVERED IN CONFIDENCE AND RELIANCE THAT SUCH INFORMATION WILL NOT BE COPIED OR MADE AVAILABLE TO ANY THIRD PARTY WITHOUT THE WRITTEN CONSENT OF SelectImmune PHARMA AB.

List Of Abbreviations

AE	Adverse Event
ALP	Alkaline Phosphatase
AUC	Area Under Curve
CA	Competent Authority
CDMS	Clinical Data Management System
cGMP	Current Good Manufacturing Practice
CI	Confidence Interval for mean
CMO	Contract Manufacturing Organisation
CRF	Case Report Form
CRO	Contract Research Organisation
CTP	Clinical Trial Protocol
CTCAE	Common Terminology Criteria for Adverse Events
DRESS	Drug reaction with eosinophilia and systemic symptoms
EDC	Electronic Data Capture
EU	European Union
FSI	First Subject In
GCP	Good Clinical Practice
GDPR	General Data Protection Regulation
IB	Investigator´s Brochure

THIS DOCUMENT CONTAINS TRADE SECRETS, AND/OR COMMERCIAL INFORMATION, DELIVERED IN CONFIDENCE AND RELIANCE THAT SUCH INFORMATION WILL NOT BE COPIED OR MADE AVAILABLE TO ANY THIRD PARTY WITHOUT THE WRITTEN CONSENT OF SelectImmune PHARMA AB.

ICH	International Conference on Harmonisation
ID	Identification
IMP	Investigational Medicinal Product
IEC	Independent Ethics Committee
IL-1β	Interleukin-1beta
IRB	Institutional Review Board
LSO	Last Subject Out
NK1R	Neurokinin-1 receptor
RA	Rheumatoid Arthritis
SAE	Serious Adverse Event
SJIA	Systemic Juvenile Idiopathic Arthritis
SmPC	Summary of Product Characteristics
SOP	Standard Operating Procedure
SP	Substance P
SPM	Study Procedures Manual
SUSAR	Suspected Unexpected Serious Adverse Reaction
WMA	World Medical Association

THIS DOCUMENT CONTAINS TRADE SECRETS, AND/OR COMMERCIAL INFORMATION, DELIVERED IN CONFIDENCE AND RELIANCE THAT SUCH INFORMATION WILL NOT BE COPIED OR MADE AVAILABLE TO ANY THIRD PARTY WITHOUT THE WRITTEN CONSENT OF SelectImmune PHARMA AB.

Synopsis

**Title of the Trial**	Immunomodulatory treatment of recurrent cystitis
**Sponsor**<br><br>**Contractor**	**SelectImmune Pharma AB, Lund (Sweden)**<br><br>Coordinating Center for Clinical Trials of the Philipps-University of Marburg with a branch office at the Justus-Liebig-University Giessen<br>Klinikstrasse 29<br>35392 Giessen, Germany
**Coordinating Investigator**<br>**(LKP according to §40 AMG)**	Dr Florian Wagenlehner, MD, Professor, Justus Liebig University of Giessen, Germany<br>Clinic and Polyclinic for Urology, Pediatric Urology and Andrology<br>Rudolf-Buchheim-Strasse 7, 35392 Giessen, Germany
**Investigational Products**	Kineret® and Nitrofurantoin Uro-Tablinen®
**Indication**	Uncomplicated recurrent lower urinary tract infection in female patients
**Objectives**	Primary Efficacy Objective:<br>To investigate treatment efficacy of Kineret® compared to Nitrofurantoin Uro-Tablinen® in female patients with an acute episode of recurrent cystitis.<br><br>a) To investigate whether Kineret® is effective in treatment of acute cystitis.<br><br>b) To investigate whether Kineret® treatment decreases the incidence of recurrent cystitis.<br><br>Secondary Efficacy Objective:<br>a) To define the molecular mechanisms resulting in recurrent cystitis, based on animal data.<br><br>b) To map the molecular effects of Kineret® in patients with recurrent cystitis.<br><br>Safety Objective:<br>To evaluate the safety and tolerability of the study treatments.
**Study Design**	An open label, randomized, single-center, 2-arm parallel group, phase II trial.

THIS DOCUMENT CONTAINS TRADE SECRETS, AND/OR COMMERCIAL INFORMATION, DELIVERED IN CONFIDENCE AND RELIANCE THAT SUCH INFORMATION WILL NOT BE COPIED OR MADE AVAILABLE TO ANY THIRD PARTY WITHOUT THE WRITTEN CONSENT OF SelectImmune PHARMA AB.

	Randomisation is carried out in a 2:1 ratio into the two treatment groups - Kineret® or Nitrofurantoin Uro-Tablinen®
**Number of Patients**	In total 30 patients will be recruited. 20 patients will be randomised to Kineret®and 10 patients will be randomised to Nitrofurantoin Uro-Tablinen®
**Eligibilty Criteria - Inclusion**	• Stable patients with an acute episode of recurrent uncomplicated cystitis.<br>• Signed written informed consent form<br>• Capability and willingness to comply with study procedures<br>• Female subjects, 18 - 65 years old<br>• Negative urine pregnancy test in women of childbearing potential.<br>• Sum score of the main uUTI symptoms reported on the Acute Cystitis Symptom Score (ACSS) typical domain at visit 1 is ≥ 6<br>• Typical symptoms must be acute, developed within ≤ 6 days<br>• Historys of recurrent cystitis (≥ 3 documented episodes in the history in the past year) or (≥ 2 documented episodes in the history in the past 6 months))<br>• Contraception<br>Contraception should be maintained during treatment and 30 days after last intake. Women of childbearing potential must use contraceptional methods which are considered as highly effective birth control methods. Such methods include:<br>- combined (estrogen and progestogen containing) hormonal contraception<br>associated with inhibition of ovulation:<br>• oral<br>• intravaginal<br>• transdermal<br>- progestogen-only hormonal contraception<br>associated with inhibition of ovulation:<br>• oral<br>• injectable<br>• implantable<br>- intrauterine device (IUD)<br>- intrauterine hormone-releasing system (IUS)<br>- bilateral tubal occlusion<br>- vasectomised partner<br>- sexual abstinence<br>Hormonal contraception methods should be combined with barrier methods.

THIS DOCUMENT CONTAINS TRADE SECRETS, AND/OR COMMERCIAL INFORMATION, DELIVERED IN CONFIDENCE AND RELIANCE THAT SUCH INFORMATION WILL NOT BE COPIED OR MADE AVAILABLE TO ANY THIRD PARTY WITHOUT THE WRITTEN CONSENT OF SelectImmune PHARMA AB.

**Eligibility Criteria - Exclusion**	• Breastfeeding<br>• Signs of pyelonephritis<br>• Uncontrolled Diabetes Mellitus<br>• Neutropenia (<1,5x10^9/L)<br>• Signs of genital infections (Vaginitis/ cervizitis)<br>• Anamnestic exclusion of patients with active severe infections, history of viral hepatitis (only anamnestic exclusion possible, as immediate inclusion in the study is urgently required for this disease pattern. Serological and other blood test results (processing time ≥ 24 hours) cannot be waited for).<br>• Patients using CYP450 medications (e.g. Warfarin, Phenytoin)<br>• Patients using Phenytoin medication<br>• Anamnestic exclusion of patients with a history of tuberculosis or active tuberculosis (only anamnestic exclusion possible, as immediate inclusion in the study is urgently required for this disease pattern. Serological blood test results (processing time ≥ 24 hours) cannot be waited for)<br>• Extragenital conditions, nephrological conditions, urological conditions, urinary catheters that may lead to complicated UTI<br>• Severe uncontrolled systemic disease<br>• Systemic Antibiotic therapy within 5 days prior to inclusion<br>• Impaired renal function (Creatinine clearance < 60 ml /min)<br>• Known allergies/contraindications to Kineret® and Nitrofurantoin Uro-Tablinen®<br>• Malign diseases<br>• Immunosuppression<br>• Previously enrolled in this trial<br>• Pathological liver enzymes<br>• Polyneuropathies<br>• Glucose-6-phosphate dehydrogenase deficiency<br>• Participation in other interventional clinical trials<br>• Use of Diclofenac and Ibuprofen analgetic medication while participating in this clinical trial

THIS DOCUMENT CONTAINS TRADE SECRETS, AND/OR COMMERCIAL INFORMATION, DELIVERED IN CONFIDENCE AND RELIANCE THAT SUCH INFORMATION WILL NOT BE COPIED OR MADE AVAILABLE TO ANY THIRD PARTY WITHOUT THE WRITTEN CONSENT OF SelectImmune PHARMA AB.

**Investigational Product**<br>**Dosing**<br>**Administration**<br><br>**Control Medication**<br>**Dosing**<br><br>**Administration**	Kineret®<br>100 mg q.d. at Days 1,2,3,4,5<br>Subcutaneous injection<br><br>Nitrofurantoin Uro-Tablinen® (retard effect, 50 mg)<br>100 mg, two times per day over 5 days (adapted to patient weight – daily dose 3,6 mg/kg patient weight)<br>Oral
**Primary Endpoint**	Reduction in symptom score measured by the ACSS in the first 5 days (ACSS dynamics)
**Secondary Endpoints**	• Bacteriuria at visits until 26 weeks<br>• Leucocyturia at visits until 26 weeks<br>• Recurrent UTI episodes until 6 months
**Safety Endpoints**	Adverse event, treatment emergent adverse events, serious adverse events
**Exploratory Efficacy Endpoints**	• Urine proteomics<br>• Gene expression analysis<br>• DNA sequence analysis
**Statistical analysis**	The statistical analysis will be specified in a Statistical Analysis Plan which will be finalised prior to Database Lock.
**Expected trial duration**	Recruitment period (months): 12<br>First patient in to last patient out (months): 18
**Translational research**	Molecular data regarding disease susceptibility is translated into the clinic. A combination of molecular and clinical parameters will be tested. The outcome from this trial will help answer the critical need of finding novel compounds to combat antibiotic resistant bacterial infections.

THIS DOCUMENT CONTAINS TRADE SECRETS, AND/OR COMMERCIAL INFORMATION, DELIVERED IN CONFIDENCE AND RELIANCE THAT SUCH INFORMATION WILL NOT BE COPIED OR MADE AVAILABLE TO ANY THIRD PARTY WITHOUT THE WRITTEN CONSENT OF SelectImmune PHARMA AB.

1 Trial Identification

The EudraCT number for this protocol is 2019-004209-28. The protocol code number is SP001-001.

2 Introduction and Rationale

2.1 Background

Urinary tract infections (UTI) are one of the most common infectious diseases in the world, affecting approximately 150 million people each year. The most common form of UTI is bacterial cystitis, which mainly affects women and is frequently recurring. A history of acute cystitis constitutes the greatest risk factor for recurrent cystitis. The global epidemic of antibiotic resistant bacteria is alarming, making these infections increasingly difficult to treat and the need for treatment alternatives quite urgent. WHO describes antibiotic resistance as one of the biggest threats to human health globally.

In recent years, new knowledge has defined molecular mechanisms and partly explained the link between infection, inflammation and pain in the bladder. Our research has shown that acute cystitis is caused by hyper-inflammation of the bladder and surrounding structures caused by Interleukin-1, which drives the disease process. As a result, blockade of the IL-1 receptor (IL-1R) with an IL-1R antagonist (Anakinra) protects against acute cystitis in animal models. In animal models, we have also defined how IL-1 overactivation occurs molecularly and how IL-1 drives the inflammatory process in the lower urinary tract [1]. It has also been shown in a clinical study that patients with acute cystitis have elevated levels of IL-1 in urine [1]. These findings illustrate for the first time the immunological background to acute cystitis and demonstrate Kineret's potential for the treatment of acute cystitis in humans.

Kineret® (Anakinra) is a recombinant IL-1R antagonist that has been approved for clinical use to treat various rheumatological diseases [2]. Treatment is chronic in many cases and well tolerated with an extensively documented safety profile. In exceptional cases, treatment may increase the risk of infections, primarily within the respiratory tract [3-5]. We have not found any support in the literature that prolonged Kineret® treatment would cause any risk of UTI.

This is in good agreement with our research results identifying specific immunological mechanisms linked to UTI and specifically acute cystitis. In an animal model, Kineret® was shown to inhibit the inflammatory process of the bladder during acute cystitis [1]. The inhibited inflammatory response in turn lead to decreased bacterial levels, an effect comparable to antibiotic therapy, and partly to rapidly decreasing symptoms.

THIS DOCUMENT CONTAINS TRADE SECRETS, AND/OR COMMERCIAL INFORMATION, DELIVERED IN CONFIDENCE AND RELIANCE THAT SUCH INFORMATION WILL NOT BE COPIED OR MADE AVAILABLE TO ANY THIRD PARTY WITHOUT THE WRITTEN CONSENT OF SelectImmune PHARMA AB.

Interest in the use of anti-inflammatory drugs has increased in recent years, mainly in the form of NSAIDs, as an alternative to antibiotic therapy for uncomplicated cystitis with varying results [6]. The studies show that NSAIDs can be useful for treatments of acute symptoms and may slightly decrease recurrent cystitis, but due to a lack of specificity, these anti-inflammatory agents cannot completely replace antibiotics in the treatment of acute cystitis [7-9]. We believe that our strategy, to block the IL-1 receptor, has a much more precise and well-defined molecular mechanism as well as extensive support in experimental models.

Based on the research results described above, we want to evaluate the possibility of treating recurrent acute cystitis with Kineret. We want to investigate whether the treatment provides adequate cure for acute cystitis compared to conventional antibiotic treatment, both based on the patient's symptoms and inflammation and on the effects on bacteria in the urine.

2.2 Clinical experience with Kineret

Rheumatoid Arthritis (RA)

Kineret has been used for years in combination with methotrexate for the treatment of symptoms in adults with RA who do not respond adequately to methotrexate alone. The recommended dose of Kineret is 100 mg administered subcutaneously once daily. The dose should be given at approximately the same time each day. Kineret is ready for use in a graduated pre-filled syringe. The graduated pre-filled syringe allows doses between 20 and 100 mg.

Pharmacokinetics

The absolute bioavailability of Kineret following a 70 mg subcutaneous bolus injection in healthy subjects (n = 11) is 95%. After a subcutaneous injection, the absorption process is the rate limiting factor for the elimination of plasma Kineret. In rheumatoid arthritis subjects, maximal plasma concentrations of Kineret were reached 3 to 7 hours after subcutaneous administration of Kineret at clinically relevant doses (1 to 2 mg/kg; n = 18). Plasma concentration decreased without any noticeable distribution phase and the elimination half-life was between 4 and 6 hours. In patients with rheumatoid arthritis, no unexpected accumulation of Kineret was observed after daily subcutaneous doses for up to 24 weeks. Mean (SD) estimates of clearance (CL / F) and volume of distribution (Vd / F) by population analysis of data from two PK studies in 35 patients with rheumatoid arthritis were 105 (27) ml / min and 18.5 (11) liters, respectively [10, 11]. Data from humans and animal data showed that the

THIS DOCUMENT CONTAINS TRADE SECRETS, AND/OR COMMERCIAL INFORMATION, DELIVERED IN CONFIDENCE AND RELIANCE THAT SUCH INFORMATION WILL NOT BE COPIED OR MADE AVAILABLE TO ANY THIRD PARTY WITHOUT THE WRITTEN CONSENT OF SelectImmune PHARMA AB.

elimination of Kineret is mainly via the kidneys. Clearance of Kineret in patients with rheumatoid arthritis increased with increased creatinine clearance.

The effect of demographic differences on the pharmacokinetics of Kineret was studied by pharmacokinetic analysis of the population of 341 patients who received daily subcutaneous injections of Kineret at doses of 30, 75 and 150 mg for up to 24 weeks [10, 12]. The estimated clearance of Kineret increased with increased creatinine clearance and body weight. The pharmacokinetic analysis of the population showed that the mean plasma clearance after subcutaneous bolus administration was approximately 14% higher for men than for women and approximately 10% higher for subjects <65 years compared to subjects ≥ 65 years. After adjusting for creatinine clearance and body weight, gender and age were not significant factors for the mean plasma clearance. No dose adjustment is required based on age or gender.

2.3 Pre-clinical findings

An *in vivo* study published in 2016 identified acute cystitis as an Interleukin-1beta (IL-1β)-driven, hyper-inflammatory condition of the infected urinary bladder and IL-1 receptor blockade as a novel therapeutic strategy. In the study, the disease severity was controlled by the mechanism of IL-1β processing and mice with intact inflammasome function developed a moderate, self-limiting form of cystitis [1]. A second study showed that infection activates Neurokinin-1 receptor (NK1R) and Substance P (SP) expression in nerve cells and bladder epithelial cells *in vitro* and *in vivo* in the urinary bladder mucosa by an IL-1β driven mechanism [13]. The suggested clinical relevance was supported by elevated urine SP levels in patients with acute cystitis, compared to patients with asymptomatic bacteriuria. Blocking of IL-1β receptor was proposed to control pain and mucosal inflammation [13].

2.4 Trial Rationale

2.4.1 Overall Trial Rationale

The overall rationale of this pilot trial is to investigate whether Kineret may offer patients with recurrent acute cystitis an alternative treatment to that of antibiotics. Due to the increased development of resistance to antibiotics, there is a need of alternative treatments.

Acute cystitis is common and the disease burden is especially great in patients with recurrent infections, which can result in a markedly reduced quality of life and debilitating pain syndromes. This study is based on novel findings that define the mechanism of acute cystitis, including the inflammatory signalling cascades that are activated by the bacteria and the host

THIS DOCUMENT CONTAINS TRADE SECRETS, AND/OR COMMERCIAL INFORMATION, DELIVERED IN CONFIDENCE AND RELIANCE THAT SUCH INFORMATION WILL NOT BE COPIED OR MADE AVAILABLE TO ANY THIRD PARTY WITHOUT THE WRITTEN CONSENT OF SelectImmune PHARMA AB.

immune defects that allow infection to occur and recur in specific individuals. Effective treatment was achieved in an animal model, using Kineret [1, 13]. This included mice with genetic imuno-deficiencies that would have suffered from very severe disease if not treated. Importantly, this treatment both reduced inflammation in the tissue and improved bacterial clearence, a combination which would be extremely important to achieve in the clinic, not least in patients with infections caused by antibiotic resistant strains.

2.4.2 Rationale for safety

The clinical safety and tolerability of Kineret administration has been well documented as Kineret is a registered drug. In 2002 Kineret received a marketing authorisation valid througout the European Union. Kineret is used to treat various rheumatological diseases and treatment is in many cases chronic. The safety profile of Kineret has thus been studied extensively in clinical use as well as in a large number of placebo controlled studies. The side effects of Kineret have been documented to be mostly mild [10].

2.4.3 Rationale for efficacy

Kineret is a recombinant human IL-1 receptor blocker. This molecule has been used for many years in the treatment of arthritis, but also in other diseases where acute inflammation causes harm to the patient. We could see that Kineret has shown similar efficacy as antibiotics in an animal cystitis model that mimics human disease quite closely [13]. From our studies in mice, we observed that inhibition of uncontrolled inflammation cures urinary tract infection faster and without organ damage [1, 13]. In this trial we will follow well established guidelines for antibiotic therapy, such as the German AWMF S3 guideline management of uncomplicated UTI (AWMF 043/044)

(https://www.awmf.org/uploads/tx_szleitlinien/043-044k_S3_Harnwegsinfektionen_2017-05.pdf).

Recurrent acute cystitis occurs in a subset of patients with elevated susceptibility, possibly due to a less effective immune system. Kineret will be used as a way of compensating this defect in the patients and improve the clearance of infection.

2.4.4 Rationale for female patients

The disease uncomplicated (recurrent) cytitis occurs only in women. Therefore, only female patients are included in this clinical trial.

THIS DOCUMENT CONTAINS TRADE SECRETS, AND/OR COMMERCIAL INFORMATION, DELIVERED IN CONFIDENCE AND RELIANCE THAT SUCH INFORMATION WILL NOT BE COPIED OR MADE AVAILABLE TO ANY THIRD PARTY WITHOUT THE WRITTEN CONSENT OF SelectImmune PHARMA AB.

2.4.5 Rationale for lifestyle considerations

Since CYP450 food products like grapefruit and herbal products like St. John`s wort may interact with the study medication, patients are instructed to waive such products while participating in this clinical trial.

2.5 Ethical and Legal Consideration Statement

The information in this clinical trial protocol (CTP) is consistent with current knowledge of the risks and benefits of the study drugs as well as the ethical and scientific principles set out in the Declaration of Helsinki and the guidelines for Good Clinical Practice (GCP). The protocol is thus designed to ensure that the Sponsor, its designee and site staff conduct the trial according to the ethical and scientific principles governing clinical research.

The investigator must ensure that patients' anonymity will be maintained and that their identities are protected from unauthorized parties in accordance with General Data Protection Regulation (GDPR) requirements.

Competent Authority (CA) approval as well as approval of the Independent Ethics Committee (IEC) must be in place prior to trial start.

Essential amendment to the trial protocol will not be implemented by either the Sponsor or the Investigator without agreement by both parties and prior to written approval by CA and IEC as required. Exceptions to this is a change of set protocol procedures to eliminate immediate hazards to participating patients. A protocol amendment will then retrospectively be submitted to the CA/IEC for approval. Any deviation from the protocol must be fully explained and documented by the Investigator.

The Investigator must sign the affiliated Protocol Signature Page. The signature confirms that the Investigator has reviewed, agreed and approved the requirements contained in this protocol.

2.5.1 Potential Risks and Benefits

The study drug Kineret is well tested in patients with rheumatoid arthritis and a large number of placebo-controlled studies have shown Kineret to be well-tolerated. Kineret is a registered drug for patients with rheumatoid arthritis thus the side effects of Kineret are well known. The most common side effects with Kineret are headache, injection site reactions and increase in cholesterol levels. The full list of side effects is listed in the SmPc of the drug.

THIS DOCUMENT CONTAINS TRADE SECRETS, AND/OR COMMERCIAL INFORMATION, DELIVERED IN CONFIDENCE AND RELIANCE THAT SUCH INFORMATION WILL NOT BE COPIED OR MADE AVAILABLE TO ANY THIRD PARTY WITHOUT THE WRITTEN CONSENT OF SelectImmune PHARMA AB.

The inclusion and exclusion criteria for the present trial have been designed to exclude patients for whom treatment with Kineret is contradicted. The risks that the patient randomized to Kineret is exposed to in connection with the study are therefore considered small.

Patients that participate may still be exposed to potential risks in the form of unexpected side effects. Adverse events and side effects will be collected throughout the course of the study, partly through spontaneous reporting by the research person and by the Investigator and delegated study staff that gather information at the regular visits of the patients. This will enable appropriate actions to be taken to maintain patients' safety.

Potential benefits of Kineret treatment are the anticipated clinical effect to improve signs and symtoms of infection and to eradicate the infection. If Kineret is proven to be effective, patients with recurrent cystitis may have future access to better alternative treatments and improved preventive measures could also be the result. Participation in this trial is voluntary and subjects are allowed to withdraw at any time.

Participants in the trial will be under careful supervision by a qualified Investigator during the entire trial period. If any potentially serious reactions are observed, subjects will be withdrawn at the Investigator's discretion or according to withdrawal criteria (Section 5.6.1).

The risk-benefit of Kineret treatment for patients with recurrent cystitis is judged to be favourable.

Patients randomised to the control group will be treated with conventional antibiotics used for this group of patients and will not, due to the participation in this trial, be exposed to further risks than in routine praxis.

3 Trial Objectives and Endpoints

The overall objective of the trial is to evaluate efficacy and safety of Kineret. Kineret will be investigated for treatment of episodes of acute cystitis in female patients with a history of recurrent cystitis, i.e. recurrent episodes of lower urinary tract infections. Kineret will be compared to conventional antibiotic treatment with Nitrofurantoin, using a randomized protocol.

THIS DOCUMENT CONTAINS TRADE SECRETS, AND/OR COMMERCIAL INFORMATION, DELIVERED IN CONFIDENCE AND RELIANCE THAT SUCH INFORMATION WILL NOT BE COPIED OR MADE AVAILABLE TO ANY THIRD PARTY WITHOUT THE WRITTEN CONSENT OF SelectImmune PHARMA AB.

3.1 Objectives

3.1.1 Primary Efficacy Objective

To investigate treatment efficacy of Kineret compared to Nitrofurantoin in female patients with an acute episode of recurrent cystitis.

a) To investigate whether Kineret is effective in treatment of acute cystitis.

b) To investigate whether Kineret treatment decreases the incidence of recurrent cystitis.

3.1.2 Secondary Efficacy Objectives

a) To define the molecular mechanisms resulting in recurrent cystitis, based on animal data.

b) To map the molecular effects of Kineret in patients with recurrent cystitis.

3.1.3 Safety Objective

To evaluate the safety and tolerability of the study treatments.

3.2 Endpoints

3.2.1 Primary Efficacy Endpoint

Reduction in symptom score measured by the ACSS in the first 5 days (ACSS dynamics).

3.2.2 Safety Endpoints

Change in laboratory safety variables, vital signs, physical examination from baseline and the incidence of adverse events, treatment emergent adverse events, serious adverse events during the trial.

3.2.3 Secondary Endpoints

a) Presence of bacteriuria at each visit up to week 26 of study
b) Presence of leucocyturia at each visit up to week 26 of study
c) Recurrent UTI episodes up to 6 months
d) Urine proteomics
e) Gene expression analysis
f) DNA sequence analysis

THIS DOCUMENT CONTAINS TRADE SECRETS, AND/OR COMMERCIAL INFORMATION, DELIVERED IN CONFIDENCE AND RELIANCE THAT SUCH INFORMATION WILL NOT BE COPIED OR MADE AVAILABLE TO ANY THIRD PARTY WITHOUT THE WRITTEN CONSENT OF SelectImmune PHARMA AB.

3.2.4 Exploratory Efficacy Endpoints

a) Urine proteomics
b) Gene expression analysis
c) DNA sequence analysis

4 Trial Design

4.1 Overall Trial Design

Figure 1: Course of the trial

The duration of conventional treatment of acute cystitis with antibiotics is one to five days, depending on the antibiotic substance. The follow up period of 26 weeks is chosen to analyse frequency of recurrent cystitis.

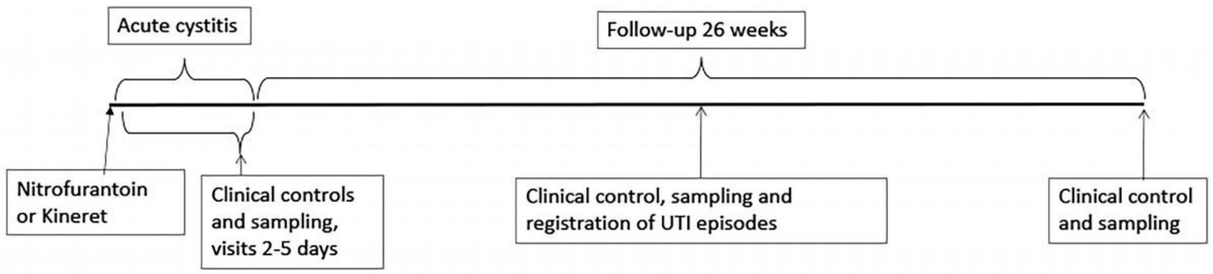

This will be a single center, randomized, open-labeled clinical trial in female subjects with a history of recurrent acute cystitis. The subjects will be randomised to receive either 5 days of Kineret treatment, 100 mg daily as a subcutaneous injection through daily visits at the study clinic or Nitrofurantoin oral antibiotic treatment for 5 days.

4.2 Number of Trial Subjects

20 subjects will be treated with Kineret treatment and 10 subjects with Nitrofurantoin. For determination of sample size see section 9.

4.3 Subject identification and randomisation of treatment

All enrolled subjects will be randomized 2:1 to two study treatment groups at Visit 1.

4.3.1 Subject identification

Subject screening numbers will be generated sequentially to subjects consenting to participate in the study and used throughout the study as subject identifier (PatientID).

THIS DOCUMENT CONTAINS TRADE SECRETS, AND/OR COMMERCIAL INFORMATION, DELIVERED IN CONFIDENCE AND RELIANCE THAT SUCH INFORMATION WILL NOT BE COPIED OR MADE AVAILABLE TO ANY THIRD PARTY WITHOUT THE WRITTEN CONSENT OF SelectImmune PHARMA AB.

4.3.2 Labelling, randomisation and treatment allocation to the study subject

Approved commercial products (e.g. Kineret and Nitrofurantoin Uro-Tablinen®) are used, which is why labeling according to §5 (8) GCP-V is waived in the context of clinical trials. Unmodified German market goods according to §5 (8) GCP-V are used in this study.

Both drugs, the verum Kineret® and the standard antibiotic Nitrofurantoin, are already approved drugs in the EU. Both are used as commodities in this study and are purchased through the local pharmacy of UKGM Marburg-Giessen. There is no change in either IMP. Patients receiving Nitrofurantoin Uro-Tablinen® will take this drug orally at home on their own twice a day (2x 100 mg). Proper documentation, including accompanying documents, will ensure that the antibiotic Nitrofurantoin is used properly.

Randomization will be performed centrally by the central office of the Center for Clinical Trials of the Philipps-University Marburg.

Randomization will not be possible during public holiday.

Koordinierungszentrum für klinische Studien (KKS)
Philipps-Universität Marburg
Karl-von-Frisch-Straße 4
D-35043 Marburg
Monday-Thursday: 8.00 - 16.00 h
Friday: 08.00 - 14.00 h
E-Mail: rando@kks.uni-marburg.de
(Fax No.: +49 (0) 6421 28 66 516 → if transmission via e-mail is not possible)

The randomization of an eligible patient can take place if all inclusion criteria and none of the exclusion criteria are fulfilled. Therefore the investigator completes the study specific randomization request form, which is part of the Investigator Site File (ISF) and sends it to the KKS Marburg via e-mail (preferably) or fax.

The chance for allocation to the verum group or control group is 2:1.

KKS Marburg will report the randomization result.

Patients randomized to Kineret will be allocated 5 vials with investigational product. Patients randomized to antibiotics will receive treatment for 5 days.

THIS DOCUMENT CONTAINS TRADE SECRETS, AND/OR COMMERCIAL INFORMATION, DELIVERED IN CONFIDENCE AND RELIANCE THAT SUCH INFORMATION WILL NOT BE COPIED OR MADE AVAILABLE TO ANY THIRD PARTY WITHOUT THE WRITTEN CONSENT OF SelectImmune PHARMA AB.

Since this is an open study the administrated Investigational product will be documented together with date, time and study code in the source documentation and eCRF.

5 Trial Population and Withdrawal

5.1 Subject Eligibility

The (sub)investigator should only enrol subjects who meet all eligibility criteria, are not put at undue risk by participating in the trial and can be expected to comply with the protocol.

The subject's eligibility for the clinical trial must be checked according to the inclusion and exclusion criteria as specified below in section 5.2. and 5.3. See also "Schedule of Trial Procedures" (section 6.1).

5.2 Inclusion Criteria for All Subjects

1. Stable patients with an acute episode of recurrent uncomplicated cystitis.
2. Signed written informed consent form
3. Capability and willingness to comply with study procedures
4. Female subjects, 18 - 65 years old
5. Negative urine pregnancy test in women of childbearing potential.
6. Sum score of the main uUTI symptoms reported on the Acute Cystitis Symptom Score (ACSS) typical domain at visit 1 is ≥ 6
7. Typical symptoms must be acute, developed within ≤ 6 days
8. Historys of recurrent cystitis (≥ 3 documented episodes in the history in the past year) or (≥ 2 documented episodes in the history in the past 6 months))
9. Contraception

Contraception should be maintained during treatment and 30 days after last intake. Women of childbearing potential must use contraceptional methods which are considered as highly effective birth control methods. Such methods include:

- combined (estrogen and progestogen containing) hormonal contraception associated with inhibition of ovulation:

  - oral
  - intravaginal
  - transdermal

THIS DOCUMENT CONTAINS TRADE SECRETS, AND/OR COMMERCIAL INFORMATION, DELIVERED IN CONFIDENCE AND RELIANCE THAT SUCH INFORMATION WILL NOT BE COPIED OR MADE AVAILABLE TO ANY THIRD PARTY WITHOUT THE WRITTEN CONSENT OF SelectImmune PHARMA AB.

- progestogen-only hormonal contraception associated with inhibition of ovulation:
  - oral
  - injectable
  - implantable

- intrauterine device (IUD)

- intrauterine hormone-releasing system (IUS)

- bilateral tubal occlusion

- vasectomised partner

- sexual abstinence

Hormonal contraception methods should be combined with barrier methods.

5.3 Exclusion Criteria for All Subjects

1. Breastfeeding
2. Signs of pyelonephritis
3. Uncontrolled Diabetes Mellitus
4. Neutropenia (<1,5x10^9/L)
5. Signs of genital infections (Vaginitis/ cervizitis)
6. Anamnestic exclusion of patients with active severe infections, history of viral hepatitis (only anamnestic exclusion possible, as immediate inclusion in the study is urgently required for this disease pattern. Serological and other blood test results (processing time ≥ 24 hours) cannot be waited for).
7. Patients using CYP450 medications (e.g. Warfarin, Phenytoin)
8. Patients using Phenytoin medication
9. Anamnestic exclusion of patients with a history of tuberculosis or active tuberculosis (only anamnestic exclusion possible, as immediate inclusion in the study is urgently required for this disease pattern. Serological blood test results (processing time ≥ 24 hours) cannot be waited for)
10. Extragenital conditions, nephrological conditions, urological conditions, urinary catheters that may lead to complicated UTI
11. Severe uncontrolled systemic disease
12. Systemic Antibiotic therapy within 5 days prior to inclusion
13. Impaired renal function (Creatinine clearance < 60 ml /min)

THIS DOCUMENT CONTAINS TRADE SECRETS, AND/OR COMMERCIAL INFORMATION, DELIVERED IN CONFIDENCE AND RELIANCE THAT SUCH INFORMATION WILL NOT BE COPIED OR MADE AVAILABLE TO ANY THIRD PARTY WITHOUT THE WRITTEN CONSENT OF SelectImmune PHARMA AB.

14. Known allergies/contraindications to Kineret and Nitrofurantoin
15. Malign diseases
16. Immunosuppression
17. Previously enrolled in this trial
18. Pathological liver enzymes
19. Polyneuropathies
20. Glucose-6-phosphate dehydrogenase deficiency
21. Participation in other interventional clinical trials
22. Use of Diclofenac and Ibuprofen analgetic medication while participating in this clinical trial

5.4 Subject Enrolment Log

Investigators must keep a list of all subjects who are considered for enrolment into the study even if they are not subsequently enrolled.The subjects will be listed on a Subject Pre-Screening- / Screening Log. This log will list whether subjects were enrolled or not. If a subject is not enrolled, the main reason will be listed. The list will be prepared according to GDPR.

5.5 Subject Identification List

The investigator must also keep a confidential Subject Identification List of all subjects entered to treatment linking the subject’s identifier (PatientID) to each subject’s full identity to enable quality control of data and allow the Investigator to quickly identify a subject when needed. The list is kept by the investigator and must not be copied or retained by SelectImmune Pharma or KKS.

5.6 Discontinuation Criteria

5.6.1 Withdrawal of patients from study treatment

All subjects have the right to withdraw from the study at any time, for any reason, and without penalty or loss of benefits to which the patient is otherwise entitled. If the patient chooses to withdraw, the investigator must be informed immediately.

Treatment with study medication may also be terminated by the investigator for several reasons e.g.

THIS DOCUMENT CONTAINS TRADE SECRETS, AND/OR COMMERCIAL INFORMATION, DELIVERED IN CONFIDENCE AND RELIANCE THAT SUCH INFORMATION WILL NOT BE COPIED OR MADE AVAILABLE TO ANY THIRD PARTY WITHOUT THE WRITTEN CONSENT OF SelectImmune PHARMA AB.

- Any signs and symptoms considered by the investigator to be a safety issue
- Emergence of an unacceptable SAE/AE
- Any reason if it is in the best interest of the patient
- If the patient does not comply with investigational requirements.

Study treatment must be terminated for one of the following reasons:

- Withdrawal of patient's consent to study treatment
- Pregnancy
- Any other development in the patient's medical condition that has an unfavorable effect on the risk-benefit assessment for this individual as determined by the investigator.

If the investigator terminates the treatment with study medication prematurely, further medical treatment will be decided individually between the investigator and the patient.

In case of premature stop of study medication or the whole study for a patient by the investigator, the investigator has to inform the patient about his decision and has to record the primary reason for termination in the patient file and in the e-CRF. Final study evaluations will be done according to the visit schedule of the study. All data before termination may be used for final analysis.

5.6.2 Premature discontinuation of the study

SelectImmune Pharma, the investigator, the IRB/IECs or competent authorities may decide to stop the trial, part of the trial or a trial site at any time. Procedures to be followed must be agreed.

If a trial is suspended or prematurely terminated, the investigator must inform the subjects promptly and ensure appropriate therapy and follow-up. As specified by applicable regulatory requirements, either the investigator or SelectImmune Pharma must promptly inform IRB/IECs and provide a detailed written explanation. Relevant competent authorities must be informed.

The trial must be terminated if the perception of the benefit/risk ratio (judged from clinical signs and symptoms, (S)AEs and/or remarkable safety laboratory changes) becomes unfavourable for the continuation of the trial.

THIS DOCUMENT CONTAINS TRADE SECRETS, AND/OR COMMERCIAL INFORMATION, DELIVERED IN CONFIDENCE AND RELIANCE THAT SUCH INFORMATION WILL NOT BE COPIED OR MADE AVAILABLE TO ANY THIRD PARTY WITHOUT THE WRITTEN CONSENT OF SelectImmune PHARMA AB.

Possible reasons for termination of the study could be but are not limited to:

- Unexpected accumulation of SAE/AE
- Major failure to adhere to the study protocol
- Inaccurate or incomplete enrolment or data collection

6 Trial Schedule and Assessments

6.1 Schedule of Trial Procedures

Study chart (examples)

THIS DOCUMENT CONTAINS TRADE SECRETS, AND/OR COMMERCIAL INFORMATION, DELIVERED IN CONFIDENCE AND RELIANCE THAT SUCH INFORMATION WILL NOT BE COPIED OR MADE AVAILABLE TO ANY THIRD PARTY WITHOUT THE WRITTEN CONSENT OF SelectImmune PHARMA AB.

	Screening Visit 1	Visits 2 and 5	Visits 3 and 4	Visit 6	Safety Phone Call	Visit 7 *	Unscheduled visits
**Day**	Day 1	Day 2 and 5	Day 3 and 4	Day 15±2 days	Day 30	Week 26±5 days	
**Patients**	All patients	All patients	Only patients of Kineret® Arm	All patients	All patients	All patients	All patients
**Visit window**							
Informed consent	X						
Subject demographics	X						
In-/exclusion criteria	X						
Anamnestic exclusion of patients with active severe infections, history of viral hepatitis	X						
Anamnestic assessment of tuberculosis history or active tuberculosis	X						
Urine Pregnancy test	X					X	
Medical history and concurrent diagnosis.	X						
Concomitant medication	X	X	X	X	X	X	X
Physical examination	X					X	
Randomisation	X						

THIS DOCUMENT CONTAINS TRADE SECRETS, AND/OR COMMERCIAL INFORMATION, DELIVERED IN CONFIDENCE AND RELIANCE THAT SUCH INFORMATION WILL NOT BE COPIED OR MADE AVAILABLE TO ANY THIRD PARTY WITHOUT THE WRITTEN CONSENT OF SelectImmune PHARMA AB.

	**Screening Visit 1**	**Visits 2 and 5**	**Visits 3 and 4**	**Visit 6**	**Safety Phone Call**	**Visit 7 ***	**Unscheduled visits**
**Day**	Day 1	Day 2 and 5	Day 3 and 4	Day 15±2 days	Day 30	Week 26±5 days	
**Patients**	All patients	All patients	Only patients of Kineret® Arm	All patients	All patients	All patients	All patients
Dispensing of Study Medication (Patient randomised to Kineret®)	X	X	X				
Dispensing of study medication (Patient randomised to Nitrofurantoin Uro-Tablinen®)	X						
Adverse Event(s)	X	X	X	X	X	X	X
Vital signs	X	X	X	X		X	X
Blood sample (haematology)	X	X	On Visit 3: only WBC & differential	Only WBC & differential		Only WBC & differential	X
Blood sample (biochemistry)	X	X					X
Urine RNA	X	X		X		X	X
Urine Proteomics	X	X		X		X	X
Urine analysis culture (dip slide, bacterial culture)	X	X	X	X		X	X

THIS DOCUMENT CONTAINS TRADE SECRETS, AND/OR COMMERCIAL INFORMATION, DELIVERED IN CONFIDENCE AND RELIANCE THAT SUCH INFORMATION WILL NOT BE COPIED OR MADE AVAILABLE TO ANY THIRD PARTY WITHOUT THE WRITTEN CONSENT OF SelectImmune PHARMA AB.

	**Screening Visit 1**	**Visits 2 and 5**	**Visits 3 and 4**	**Visit 6**	**Safety Phone Call**	**Visit 7 ***	**Unscheduled visits**
**Day**	Day 1	Day 2 and 5	Day 3 and 4	Day 15±2 days	Day 30	Week 26±5 days	
**Patients**	All patients	All patients	Only patients of Kineret® Arm	All patients	All patients	All patients	All patients
Blood sample (RNA)	X	X		X		X	X
Blood sample (DNA)	X						
Urine Flow Cytometry	X	X		X		X	X
ACSS	X	X (on day 5)		X	X	X	X

* Final visit including Early Termination Visit (see below).

THIS DOCUMENT CONTAINS TRADE SECRETS, AND/OR COMMERCIAL INFORMATION, DELIVERED IN CONFIDENCE AND RELIANCE THAT SUCH INFORMATION WILL NOT BE COPIED OR MADE AVAILABLE TO ANY THIRD PARTY WITHOUT THE WRITTEN CONSENT OF SelectImmune PHARMA AB.

6.2 Description of Trial Visits

All procedures and assessments to be performed at each visit are detailed below and in the above study chart. All data will be recorded in the Patient's hospital notes or in a worksheet, as appropriate and be reported in the eCRF.

Day 1

The subject will be considered for enrolment in the study, based on a diagnosis of cystitis. The investigator will explain the nature, purpose and risks of the trial and provide the subject with a copy of the Patient Information and Informed Consent. The patient will be asked to consider and evaluate participation in the study in accordance with the Helsinki Declaration and the local applicable laws. This visit is planned for all subjects independent of treatment arm.

Visit 1 Screening and Randomisation

- Patient Consent collected
- Demographics
- Final check for study eligibility
- Anamnestic exclusion of patients with active severe infections, history of viral hepatitis
- Anamnestic assessment of tuberculosis history or active tuberculosis
- Urine Pregnancy test
- Medical History and concurrent diagnosis
- Concurrent Medical Conditions
- Concomitant Medication
- Physical Examination
- Randomisation
- Dispensing of Kineret® (to patients randomised to Kineret®)
- Patient supplied with Nitrofurantoin Uro-Tablinen® for 5 days treatment and instructed how to take the drug to fully comply with the dosage recommendation
- Adverse Events
- Vital signs
- Blood Samples (Haematology and Biochemistry) including WBC and differential for monitoring of neutrophils granulocytes
- Urine analysis RNA
- Proteomics
- Urine analysis dip slide and bacterial culture
- Blood sample (RNA)

THIS DOCUMENT CONTAINS TRADE SECRETS, AND/OR COMMERCIAL INFORMATION, DELIVERED IN CONFIDENCE AND RELIANCE THAT SUCH INFORMATION WILL NOT BE COPIED OR MADE AVAILABLE TO ANY THIRD PARTY WITHOUT THE WRITTEN CONSENT OF SelectImmune PHARMA AB.

- Blood sample (DNA)
- Flow Cytometry urine sample
- ACSS

Visits 2 and 5 Treatment Visits

Applicable to study patients of both treatment arms.

- Concomitant Medication
- Dispensing of Kineret®
- Adverse Events
- Vital Signs
- Blood sample (haematology and biochemistry)
- Urine analysis (RNA)
- Proteomics
- Urine analysis dip slide and bacterial culture
- Blood sample (RNA)
- Flow Cytometry urine sample
- ACSS (only on visit 5)

Visits 3 and 4 Treatment Visits

Only applicable to study patients randomised to Kineret® treatment.

- Concomitant Medication
- Dispensing of Kineret®
- Adverse Events
- Vital Signs
- Blood sample for assessment WBC and differential for monitoring of neutrophils granulocytes (only on day 3)
- Urine analysis bacterial culture

Visit 6 (1st Follow-up visit)

THIS DOCUMENT CONTAINS TRADE SECRETS, AND/OR COMMERCIAL INFORMATION, DELIVERED IN CONFIDENCE AND RELIANCE THAT SUCH INFORMATION WILL NOT BE COPIED OR MADE AVAILABLE TO ANY THIRD PARTY WITHOUT THE WRITTEN CONSENT OF SelectImmune PHARMA AB.

Applicable to all study patients. 15 days (+/-2 days) after the last IMP administration, a follow-up visit will take place. Investigator will assess occurrence of any on-going or new AEs. AEs will be followed until final outcome as described in Section 7.4.

- Concomitant Medication
- Adverse Events
- Vital Signs
- Blood sample for assessment WBC and differential for monitoring of neutrophils granulocytes
- Urine analysis RNA
- Proteomics
- Urine analysis dip slide and bacterial culture
- Blood sample (RNA)
- Flow Cytometry urine sample
- ACSS

Safety telephone call at day 30

Applicable to all study patients.

- Concomitant Medication
- Adverse Events
- ACSS

Visit 7 (Final Visit)

Applicable to all study patients in week 26 (+/5 days). In addition patient premature withdrawn from the study will, if possible and if patient agrees, have the procedures and assessments performed according to this final visit.

- Urine pregnancy test
- Concomitant Medication
- Physical Examination
- Adverse Events
- Vital Signs
- Blood Samples for assessment WBC and differential for monitoring of neutrophils granulocytes
- Urine analysis RNA
- Proteomics

THIS DOCUMENT CONTAINS TRADE SECRETS, AND/OR COMMERCIAL INFORMATION, DELIVERED IN CONFIDENCE AND RELIANCE THAT SUCH INFORMATION WILL NOT BE COPIED OR MADE AVAILABLE TO ANY THIRD PARTY WITHOUT THE WRITTEN CONSENT OF SelectImmune PHARMA AB.

- Urine analysis dip slide and bacterial culture
- Blood sample (RNA)
- Flow Cytometry urine sample
- ACSS

Unscheduled Visits

During the treatment period patients treated with Kineret® or Nitrofurantoin Uro-Tablinen® will be advised to contact the investigating site in case of signs and symptoms of recurrent acute cystitis infection or any side effects. An Unscheduled visit will, at the discretion of the investigator, be scheduled. Assessments performed at this visit will be:

- Concomitant medication
- Adverse Events
- Vital Signs
- Blood sample (haematology and biochemistry)
- Urine analysis RNA
- Proteomics
- Urine analysis dip slide and bacterial culture
- Blood sample (RNA)
- Flow Cytometry urine sample
- ACSS

Patients are encouraged to report to the study center as soon as they feel symptoms of cystitis in order to record symptoms with the ACSS.
According to symptoms additional assessment may take place, at the discretion of the investigator. The visit will be documented in the Unscheduled Visit Form in the eCRF.

In addition, during the follow-up period, the patients will be advised to contact the investigating site in case of signs and symptoms of recurrent acute cystitis infection or side effects. An Unscheduled Visit will, at the discrition of the investigator, be scheduled. Assessments performed at this visit will be the same as for the study treatment visits. According to signs and symptoms additional assessment may take place, at the discretion of the investigator.The visit will be documented in the Unscheduled Visit Form in the eCRF.

THIS DOCUMENT CONTAINS TRADE SECRETS, AND/OR COMMERCIAL INFORMATION, DELIVERED IN CONFIDENCE AND RELIANCE THAT SUCH INFORMATION WILL NOT BE COPIED OR MADE AVAILABLE TO ANY THIRD PARTY WITHOUT THE WRITTEN CONSENT OF SelectImmune PHARMA AB.

Early Termination Visit

Patients are free to discontinue their participation in the trial at any time and for whatever reason without affecting their right to further treatment and to an appropriate follow-up investigation. As described in section 5.6, patients may also be discontinued from the trial at any time at the discretion of the Investigator. The date and reason for withdrawal of a patient or premature discontinuation of the trial must be documented in the eCRF and the patient's medical record. The monitor should be notified if a patient discontinues prematurely from the trial.

Patient premature withdrawn from the study will, if possible and if patient agrees, have the procedures and assessments performed according to the final visit as described above.

Patients that will develop treatment failure in either arms will be treated with alternative, antibiogram directed antibiotics, and not withdrawn from study.

6.3 Demographics

Demographic data will comprise of:

- Year of birth
- Gender
- Ethnicity/ Race

6.4 Vital Signs

Vital signs (resting blood pressure and heart rate) must be assessed.

Recording of vital signs comprises:

- Heart rate (beats per minute), supine after 5 min rest
- Blood pressure, systolic and diastolic (mmHg), supine after 5 min rest
- Body temperature
- Height
- Weight

The same arm will be used for all measurements. The arm (right or left) used for measurement will be recorded in the CRF.

THIS DOCUMENT CONTAINS TRADE SECRETS, AND/OR COMMERCIAL INFORMATION, DELIVERED IN CONFIDENCE AND RELIANCE THAT SUCH INFORMATION WILL NOT BE COPIED OR MADE AVAILABLE TO ANY THIRD PARTY WITHOUT THE WRITTEN CONSENT OF SelectImmune PHARMA AB.

Assessment of vital signs resulting in abnormal values will be repeated in order to exclude an erroneous assessment. Individual results will be classified as “normal”, “abnormal of no clinical significance” or “abnormal of clinical significance”.

6.5 Physical Examination

A general physical examination should be performed.

6.6 Medical History and Concurrent Medical Conditions

Significant medical history (specified in 6.1) within the last 12 months incl. medical history of recurrent cystitis and all concurrent medical conditions must be obtained at the screening visit.

6.7 Concomitant Medication

At each study visit/contact, the investigator should question the subject about any medication taken by the subject.

All concomitant medication, with the exception of vitamins and/or dietary supplements, administered during the time period from randomisation to last administration of study drug, are to be recorded in the eCRF. This also applies to concomitant medication administered prophylactically in anticipation of reaction to the active treatment and any medication intended to treat an AE.

A prophylactic medication is a medication administered in the absence of ANY symptom and in anticipation of a reaction to the active treatment (e.g. an anti-pyretic, corticosteroids, etc.).

Similarly, concomitant medication administered for the treatment of a SAE at any time, must be recorded on the SAE Report as applicable.

Use of non-marketed/other investigational products one month prior to Visit 1 and during the trial is not permitted.

Refer to Appendix 2 for the definition of a SAE and to Section 7.7 for the applicable reporting periods and required information.

Use of concomitant treatment must be recorded in the subject’s medical record and the eCRF detailing treatment/drug name, dose, indication and dates of start and stop.

THIS DOCUMENT CONTAINS TRADE SECRETS, AND/OR COMMERCIAL INFORMATION, DELIVERED IN CONFIDENCE AND RELIANCE THAT SUCH INFORMATION WILL NOT BE COPIED OR MADE AVAILABLE TO ANY THIRD PARTY WITHOUT THE WRITTEN CONSENT OF SelectImmune PHARMA AB.

6.8 Pregnancy Test

A urine pregnancy test must be performed at the trial site at Visit 1 (before study treatment) and Visit 7 in female subjects of childbearing potential.

6.9 Adverse Events

Adverse events must be assessed and recorded as specified in section 7.

6.10 Laboratory Assessments

6.10.1 Safety Blood Tests (hematology, biochemistry)

Samples for analysis of parameters must be taken according to the schedule of trial procedures (section 6.1) or on withdrawal from or early completion of the treatment phase of the clinical trial.

The following analyses will be performed on the venipunctured blood samples:	Parameter
Haematology	Haemoglobin<br>Haematocrit<br>Red blood cell (RBC) count<br>Mean corpuscular volume (MCV)<br>White blood cell (WBC) count, including differential count<br>Platelet count
Biochemistry (serum/plasma)	Urea<br>Creatinine<br>Sodium<br>Potassium<br>Glucose

If any laboratory results are abnormal, the investigator should follow-up the subject as clinically appropriate.

Routine analyses of blood samples (clinical chemistry) will be processed at the central laboratory of the university hospital Giessen-Marburg, location Giessen. At the end of the clinical trial, parts of the samples are shipped to the local laboratory at the Lund University, Sweden. Handling and shipment instructions are provided in laboratory manuals by the laboratory at the Lund University.

THIS DOCUMENT CONTAINS TRADE SECRETS, AND/OR COMMERCIAL INFORMATION, DELIVERED IN CONFIDENCE AND RELIANCE THAT SUCH INFORMATION WILL NOT BE COPIED OR MADE AVAILABLE TO ANY THIRD PARTY WITHOUT THE WRITTEN CONSENT OF SelectImmune PHARMA AB.

6.10.2 Safety Urine analysis

Samples for analysis of the parameters listed below must be taken as specified in the schedule of trial procedures (section 6.1) or on withdrawal from or early completion of the treatment phase of the clinical trial.

The following analyses will be performed on the mid-stream urine samples:	Parameter
Urine analysis	Bacterial culture<br><br>Leucocyte counts in uncentrifuged urine by flow cytometry<br><br>Dip slide for hematuria, leucocytes

Routine analyses of urine samples (microbiology) will be processed at the microbiology laboratory of the Clinic and Polyclinic for Urology, Pediatric Urology and Andrology, Giessen. Urine status analysis (leucocyte counts) will be carried out at the central laboratory of the university hospital Giessen-Marburg, location Giessen. Urine dip stick tests (urine analysis) and urine culture analyses will be carried out at the laboratory of the Clinic and Polyclinic for Urology, Pediatric Urology and Andrology, Giessen. At the end of the clinical trial, parts of the samples are shipped to the laboratory at the Lund University, Sweden.

THIS DOCUMENT CONTAINS TRADE SECRETS, AND/OR COMMERCIAL INFORMATION, DELIVERED IN CONFIDENCE AND RELIANCE THAT SUCH INFORMATION WILL NOT BE COPIED OR MADE AVAILABLE TO ANY THIRD PARTY WITHOUT THE WRITTEN CONSENT OF SelectImmune PHARMA AB.

6.10.3 Urine Samples for efficacy

Analyses	Parameter
Urine analysis	Bacterial culture<br><br>Leucocyte counts in uncentrifuged urine by flow cytometry<br><br>Dip slide for hematuria, leucocytes

Prior to and after treatment, urine samples will be obtained and analyzed.

Routine analyses of urine samples (microbiology) will be processed at the microbiology laboratory of the Clinic and Polyclinic for Urology, Pediatric Urology and Andrology, Giessen. Urine status analysis (leucocyte counts) will be carried out at the central laboratory of the university hospital Giessen-Marburg, location Giessen. Urine dip stick tests (urine analysis) and urine culture analyses will be carried out laboratory of the Clinic and Polyclinic for Urology, Pediatric Urology and Andrology, Giessen. At the end of the clinical trial, parts of the samples are shipped to the laboratory at the Lund University, Sweden.

6.10.4 Additional samples for laboratory analysis

Samples and extracted fractions for molecular analysis (blood RNA, blood DNA, urine RNA and urine proteomics samples) will be frozen at -80° C at the University of Giessen and shipped to the laboratory at Lund University, Sweden for analysis.

6.10.5 Clinical procedures in pandemics (COVID-19)

Clinical trials are not spared from the effects of the COVID-19 pandemic and the current (political) measures. In view of the immense restrictions on public life, the conduct of the clinical trial "Immuno-modulatory treatment of recurrent cystitis - An open label, single-center, phase II trial" is also affected. Possible relevant changes in the conduct of this clinical trial during the COVID-19 pandemic may occur. The goal is to ensure that this clinical trial continues as scheduled and that the trial data can be analyzed.

On each day of the clinical trial where patients are required to come to the trial site, a Covid-19 test will be performed upon entry to the clinic, according to the current protocol of the trial site. This is usually done at the main entrance to the trial site as a routine measure. If a negative

THIS DOCUMENT CONTAINS TRADE SECRETS, AND/OR COMMERCIAL INFORMATION, DELIVERED IN CONFIDENCE AND RELIANCE THAT SUCH INFORMATION WILL NOT BE COPIED OR MADE AVAILABLE TO ANY THIRD PARTY WITHOUT THE WRITTEN CONSENT OF SelectImmune PHARMA AB.

Covid-19 test is obtained, the patient is allowed into the study center to participate in study-specific testing.

A timely and adequate supply of study medication is provided.

The patient is instructed to contact the study center immediately (by telephone, e-mail, tele-medicine, ...) as soon as health complaints and adverse events of any kind as well as adverse effects of the study medication occur. This also applies, for example, to information about hospitalizations and complaints in connection with study-related measures as well as a "COVID-19" disease.

The investigator must be informed by patients, as described here, if they experience fever or signs of infection, such as dry cough or difficulty breathing, or if a coronavirus infection has been detected. The investigator will then discuss with patients whether to pause or postpone administration of study medication, if necessary.

Depending on the severity of the event, the investigator will decide whether the personal appearance is necessary for safety reasons, e.g. to collect further findings, or what the further procedure regarding the event will be.

Likewise, the investigator will contact the patients as soon as he/she becomes aware of events that affect their health. In doing so, he will ask for further details and coordinate the further procedure with the patients.

Furthermore, it is important to immediately inform the investigator of a possible pregnancy as a study participant.

7 Adverse Events

The following terms applies: Adverse Events (AE), Adverse Drug Reaction (ADR) Serious Adverse Events (SAE), Serious Adverse Drug Reactions (SADR) and Suspected Unexpected Serious Adverse Reactions (SUSAR). For definition see Appendix 1.

7.1 Documentation of Adverse Events

All AEs occurring after signing the informed consent form must be recorded on the electronic case report forms (eCRFs) up to 30 days after the last dose of study medication was administered.

AEs must be assessed by the investigator.

THIS DOCUMENT CONTAINS TRADE SECRETS, AND/OR COMMERCIAL INFORMATION, DELIVERED IN CONFIDENCE AND RELIANCE THAT SUCH INFORMATION WILL NOT BE COPIED OR MADE AVAILABLE TO ANY THIRD PARTY WITHOUT THE WRITTEN CONSENT OF SelectImmune PHARMA AB.

For Definitions and Details please see Appendix 2.

7.2 Other Events to be Reported

7.2.1 Pregnancy

In general, pregnant women should be excluded from clinical trials where the drug is not intended for use in pregnancy. [1] If a patient becomes pregnant during administration of the study treatment , treatment should generally be discontinued. The investigator must notify the sponsor immediately of any pregnancy after being made aware of the case. The Pregnancy Report Form is to be sent by e-mail (preferably) or fax to KKS Marburg.

Philipps-Universität Marburg
KKS Marburg
Email: sae-management@kks.uni-marburg.de
(Fax-No.: +49 (0)6421 28 66 559 → if transmission via e-mail is not possible)

The course and outcome of the pregnancy should be followed up carefully, and any abnormal outcome regarding the mother or the child should be documented on the Pregnancy Report Form and reported to the KKS Marburg.

7.3 Documentation and reporting of Serious Adverse Events (SAEs)

All SAEs occurring after signing the informed consent form must be reported up to 30 days after the last dose of study medication was administered. After that time only serious adverse reactions (events, possibly related to study medication) have to be reported. For the purpose of SAE reporting the study specific reporting form has to be used. The SAE reporting form should be completed in English.

The criteria qualifying the AE to be an SAE should be recorded in the eCRF (Appendix 2).

The investigator must notify the sponsor immediately of any SAE after being made aware of the case. The SAE reporting form is to be sent by e-mail (preferably) or fax to KKS Marburg.

Philipps-Universität Marburg
KKS Marburg

[1] Cf. European Medicines Agency, 2006: ICH Topic E8. General Considerations for Clinical Trials. Step 5, Section 3.1.4.3 a).

THIS DOCUMENT CONTAINS TRADE SECRETS, AND/OR COMMERCIAL INFORMATION, DELIVERED IN CONFIDENCE AND RELIANCE THAT SUCH INFORMATION WILL NOT BE COPIED OR MADE AVAILABLE TO ANY THIRD PARTY WITHOUT THE WRITTEN CONSENT OF SelectImmune PHARMA AB.

Email: sae-management@kks.uni-marburg.de
(Fax-No.: +49 (0)6421 28 66 559 → if transmission via e-mail is not possible)

The minimum information of an initial SAE report must include:

- EudraCT-No. (if not included in SAE Form)
- Study-ID of Sponsor (if not included in SAE Form)
- Patient-ID
- Name of reporting investigator
- Medical term of the SAE
- Name of study medication
- Causality assessment (relation between the reported event and the administration of study medication)

Furthermore the following data are required in order to complete the SAE report:

- Start date of SAE
- Start date study medication
- Date of last dose (study medication) prior to SAE
- Seriousness criteria
- End date of SAE
- SAE outcome
- Severity

Relevant follow-up information must be faxed to KKS Marburg as soon as possible. SAE-Follow-Up reports also have to be recorded on the study specific SAE reporting form.

The medical term of the SAE should be an event, reaction or diagnosis rather than a list of symptoms. Symptoms of the event can be described in the narrative field of the SAE form. It is important to enter the most appropriate medical term/event term or diagnosis in the corresponding field. **Only one medical term/event term or diagnosis** should be entered on each SAE reporting form. If more than one event is to be reported a separate SAE reporting form has to be completed.

In the case of death of a trial subject, the investigator has to provide any additional information as requested by the sponsor, the competent authority concerned or the ethics committee concerned.

THIS DOCUMENT CONTAINS TRADE SECRETS, AND/OR COMMERCIAL INFORMATION, DELIVERED IN CONFIDENCE AND RELIANCE THAT SUCH INFORMATION WILL NOT BE COPIED OR MADE AVAILABLE TO ANY THIRD PARTY WITHOUT THE WRITTEN CONSENT OF SelectImmune PHARMA AB.

7.4 End date of a Serious Adverse Event

A SAE is terminated as soon as it no longer meets the previously defined SAE criterion, e.g.:

- Results in death → SAE end date = Date of patient's death
- Life-threatening → SAE end date = Date of patient's discharge
- Hospitalisation or prolongation of existing hospitalisation → SAE end date = Date of patient's discharge
- Important medical events that may require medical or surgical intervention to prevent one of the outcomes listed in the SAE definition → SAE end date = Date when patient condition improved (no longer serious)

7.5 Exceptions from SAE Reporting

not applicable

7.6 SUSAR Reporting Procedure

All suspected adverse reactions related to an investigational medicinal product that are both unexpected and serious (SUSARs) are subject to expedited reporting. The sponsor is responsible for the prompt notification to the competent authority and ethics committee concerned as well as all investigators involved. The Sponsor has delegated to KKS to notify concerned parties.

Fatal or life-threatening SUSARs will be reported as soon as possible but no later than seven calendar days after the first knowledge of the minimum criteria for expedited reporting. Relevant follow-up information is subsequently communicated within an additional eight calendar days.

All other SUSARs will be reported as soon as possible but no later than 15 calendar days after the first knowledge of the minimum criteria for expedited reporting.[2,3]

7.7 Sponsor Reporting Responsibilities

The Sponsor is responsible for assessing whether or not an SAE is expected. The relevant reference document is the Kineret® product resume (SmPC) and the antibiotic SmPC for Nitrofurantoin Uro-Tablinen®. KKS will, on behalf of the Sponsor, notify the regulatory

---

[2] Cf. European Commission, 2011: Detailed guidance on the collection, verification and presentation of adverse event/reaction reports arising from clinical trials on medicinal products for human use ('CT-3'). Section 7.1, No. 37.

[3] Cf. Bundesministerium für Justiz, 2012: GCP-Verordnung, §13 (2) und §13 (3)

THIS DOCUMENT CONTAINS TRADE SECRETS, AND/OR COMMERCIAL INFORMATION, DELIVERED IN CONFIDENCE AND RELIANCE THAT SUCH INFORMATION WILL NOT BE COPIED OR MADE AVAILABLE TO ANY THIRD PARTY WITHOUT THE WRITTEN CONSENT OF SelectImmune PHARMA AB.

authorities and concerned investigators of all relevant safety information according to the current applicable legislation.

Notification of the IECs / IRBs about all relevant events (e.g. SUSARs) will be performed by KKS Marburg.

In compliance with applicable regulations, in the event of a SUSAR, the subject's treatment code will usually be unblinded before reporting to the competent authorities, IECs/IRBs. For reporting to investigators the treatment will, if possible, be kept blind. However since this is an open-labled study this is not applicable.

8 Investigational Product(s)

8.1 Investigational Medicinal Product (IMP) – Kineret®

Product description

Kineret® is a product approved by the European Medicines Agency (EMA) in all EU countries. Product information is managed by EMA.

(https://www.ema.europa.eu/en/medicines/human/EPAR/kineret)

Approved commercial products are used, which is why the labeling according to §5 (8) GCP-V is waived in the context of clinical trials.

Kineret® is a medicine that is used to treat signs and symptoms due to inflammatory conditions such as rheumatoid arthritis, cryopyrin-associated periodic syndromes, familial Mediterranean fever and Still's disease. Inflammation is driven by pro-inflammatory cytokines such as interleukin-1 (IL-1α and IL-1β) that binds to its receptor (interleukin-1 receptor) in order to induce inflammation. Kineret® is an interleukin-1 receptor antagonist that competitively binds and blocks interleukin-1 receptor, thereby preventing IL-1 binding. The non-clinical pharmacodynamics studies show that Kineret® efficiently inhibits the action of the cytokines IL-1α and IL-1β. These cytokines are critical mediators of inflammation and joint damage in rheumatoid arthritis, cryopyrinopathies and other IL-1 driven diseases, e.g. SJIA, AOSD, and CAPS. Anakinra has a short half-life (median 5.7 hours).

An extensive number of safety pharmacology and toxicology studies of Kineret® (all included in the original submission) were conducted covering general toxicity, reproductive toxicity, genotoxicity, carcinogenicity (tumour stimulation) and antigenicity/immunotoxicity. A series of

THIS DOCUMENT CONTAINS TRADE SECRETS, AND/OR COMMERCIAL INFORMATION, DELIVERED IN CONFIDENCE AND RELIANCE THAT SUCH INFORMATION WILL NOT BE COPIED OR MADE AVAILABLE TO ANY THIRD PARTY WITHOUT THE WRITTEN CONSENT OF SelectImmune PHARMA AB.

safety pharmacology studies (all included in original submission) were conducted in an additional number of species: mice, rats, dogs, and ex vivo guinea pig ileum. No Kineret® related effects were seen in tests of central/autonomic, analgesic activity, cardiovascular, digestive, or renal functions. Since the clinical approval of Kineret® in 2002, an excessive numbers of clinical studies has been performed to investigate pharmacokinetics, pharmacodynamics etc.

-Genotoxicity

A full set of in vitro and in vivo genotoxicity tests were conducted.

-Carcinogenicity

There are no concerns about a carcinogenic potential inherent to Kineret® based on the pharmacological mode of action and observed minimal effects on the host cell resistance studies and slight enhancement of natural killer (NK) cell activity. Thus, no further studies were considered to be needed by EMA.

-Fertility

General reproductive toxicity tests were performed. Not testet on pregnant women.

-Embryo-fetal development

In the reproductive and embryo-foetal developmental toxicity studies, the rat and the rabbit were selected as the test species.

Formulation Information

- Syringe
  Kineret® is a 100 mg / 0.67 ml solution for injection in a prefilled syringe. Each graduated pre-filled syringe contains 100 mg of Kineret® per 0.67 ml (150 mg / ml). Clear, colorless to white solution for injection that may contain some product-related semi-translucent to white amorphous particles.

8.2 Manufacturing, packaging and labelling of Kineret®

Kineret® is administered by subcutaneous injection.
The Kineret® is ready for use in a graduated pre-filled syringe. The graduated pre-filled syringe allows doses between 20 and 100 mg. Because the minimum dose is 20 mg, the syringe is not

THIS DOCUMENT CONTAINS TRADE SECRETS, AND/OR COMMERCIAL INFORMATION, DELIVERED IN CONFIDENCE AND RELIANCE THAT SUCH INFORMATION WILL NOT BE COPIED OR MADE AVAILABLE TO ANY THIRD PARTY WITHOUT THE WRITTEN CONSENT OF SelectImmune PHARMA AB.

suitable for pediatric patients with a body weight below 10 kg. Do not shake the pre-filled syringe. For instructions on use and handling, see Sustainability, Storage and Handling.

It is recommended that the injection site be varied to avoid discomfort at the injection site. Cooling of the injection site, heating the injection fluid to room temperature, use of a cooling pack (before and after injection) and use of topical glucocorticoids and post-injection antihistamines can alleviate signs and symptoms of injection site reactions.

8.2.1 Selection of dose

The recommended dose of Kineret® is 100 mg once a day, given at around the same time each day.

8.2.2 Potential Toxicity in Patients

The following safety data of Kineret® refer to SmPC; Version April 2020.

No dose-limiting toxicities were observed in clinical studies. In sepsis studies, 1,015 patients received Kineret® at doses up to 2 mg / kg / hour intravenously (~ 35 times the recommended dose in rheumatoid arthritis) over a 72-hour treatment period. The profile of adverse events in these studies generally showed no difference from that in rheumatoid arthritis studies.

Kineret® is an registered product. Please see following pages:

https://www.ema.europa.eu/en/medicines/human/EPAR/kineret

https://www.ema.europa.eu/en/documents/overview/kineret-epar-medicine-overview_en.pdf

https://www.ema.europa.eu/en/documents/rmp-summary/kineret-epar-risk-management-plan-summary_en.pdf

https://www.ema.europa.eu/en/documents/variation-report/kineret-h-c-363-ii-0056-epar-assessment-report-variation_en.pdf

Tabulated summary of adverse reactions:

The frequency of adverse reactions reflects treatment with Kineret® 100 mg administered SC.

Table 1: List of adverse drug reactions associated with Kineret®

THIS DOCUMENT CONTAINS TRADE SECRETS, AND/OR COMMERCIAL INFORMATION, DELIVERED IN CONFIDENCE AND RELIANCE THAT SUCH INFORMATION WILL NOT BE COPIED OR MADE AVAILABLE TO ANY THIRD PARTY WITHOUT THE WRITTEN CONSENT OF SelectImmune PHARMA AB.

System Organ Class	Preferred term ordered by seriousness	Frequency
Infections and parasitic diseases	Serious infections	≥ 1/100
Blood and the lymphatetic system	Neutropenia	≥ 1/100
Blood and the lymphatetic system	Trombocytopenia	≥ 1/100
Immune system	Allergic reactions, including anaphylactic reactions, Angioedema, Urticaria and Pruritus	≥ 1/1000
Central and peripheral nervous system	Headache	≥ 1/10
Liver and Bile	Increased liver enzymes	≥ 1/1000
Liver and Bile	Non-infectious hepatitis	Not known
Skin and subcutaneous	Injection-site reaction	≥ 1/10
Skin and subcutaneous	Rash	≥ 1/1000
Blood assessments	Increased cholesterol	≥ 1/10

Please note that data on adverse reactions/adverse events may change over the time. For that reason, it is necessary that the investigator is familiar with the current version of the SmPC which is filed in the Investigator site file (ISF).

8.2.3 Manufacturer of study medication

The study medication is manufactured by Swedish Orphan Biovitrum AB. It will be provided by the local pharmacy (ATC code: L04AC03, Anakinra).

8.2.4 Drug Supply

The pharmacy of the clinic will provide sites with study medication on request by KKS. After the initiation visit, the center will be provided with study medication for patients.

THIS DOCUMENT CONTAINS TRADE SECRETS, AND/OR COMMERCIAL INFORMATION, DELIVERED IN CONFIDENCE AND RELIANCE THAT SUCH INFORMATION WILL NOT BE COPIED OR MADE AVAILABLE TO ANY THIRD PARTY WITHOUT THE WRITTEN CONSENT OF SelectImmune PHARMA AB.

Study medication must be received by designated personnel at the study site. The acknowledgement of receipt has to be sent back to KKS and pharmacy via fax and has to be stored in the ISF. The receipt of medication also has to be documented on the delivery log that is filed in the ISF.

8.2.5 Handling and storage of the medication

The medication has to be handled and stored safely and properly. It has to be kept in a secured location to which only the investigator and designated site personnel have access. The study medication has to be stored in a refrigerator at 2°C - 8°C. The min/max temperature has to be documented in the temperature log found in the ISF. Kineret® must remain in the outer carton in order to protect the contents from light.

For ambulatory use, Kineret® can be removed from the refrigerator for 72 hours at a temperature not exceeding 25°C, provided the expiry date has not been passed. At the end of this period, the product must not be put back in the refrigerator but must be discarded.

Kineret® is a sterile, non-preserved solution. For single use only.

Do not shake. Allow the pre-filled syringe to reach room temperature before injection.

8.2.6 Application of the study medicine

Study medication will be administered only by an authorized person at the investigator`s site.

The administration of study medication for each patient has to be documented on the drug accounting form found in the ISF. Destruction of study medication at site can take place only after sponsor approval.

Prior to administration, the solution is visually inspected for particulate matter and discoloration. Only clear and colorless to white solutions that may contain some product-related semi-transparent to white amorphous particles should be injected.

Do not shake the product.

The presence of these particles does not affect the quality of the product.

The pre-filled syringe is for single use only. Discard any unused medicine.

THIS DOCUMENT CONTAINS TRADE SECRETS, AND/OR COMMERCIAL INFORMATION, DELIVERED IN CONFIDENCE AND RELIANCE THAT SUCH INFORMATION WILL NOT BE COPIED OR MADE AVAILABLE TO ANY THIRD PARTY WITHOUT THE WRITTEN CONSENT OF SelectImmune PHARMA AB.

Any unused medicinal product or waste material should be disposed of in accordance with local requirements.

8.3 Administration of Investigational Product Kineret®:

Route of administration	The total volume of solution is administered by subcutaneous injection
Dosing frequency	One dose at 5 occasions during the trial.
Time of day for dosing	No specific requirements
Relation of time of dosing to dietary intake	No specific requirements
Relation of time of dosing to clinical assessments	Clinical assessments will be done 1-3 hours after each instillation. Final evaluation after 5 instillations.

8.3.1 Dose and adjustment of study treatment

No individual dose adjustments will be allowed. The dose of 100 mg Kineret® will be each administered at Days 1,2,3,4,5.

8.3.2 Treatment after termination of study

The treatment after completion of the clinical trial (premature or regular) is decided individually between investigator and patient.

8.3.3 Management of toxicities of IMP

No dose-limiting toxicities were observed in clinical studies. In sepsis studies, 1,015 patients received Kineret® at doses up to 2 mg / kg / hour intravenously (~ 35 times the recommended dose in rheumatoid arthritis) over a 72-hour treatment period. The profile of adverse events in these studies generally showed no difference from that in rheumatoid arthritis studies.

Drug reaction with eosinophilia and systemic symptoms (DRESS) has been rarely reported in patients treated with Kineret, especially in patients with systemic juvenile idiopathic arthritis (SJIA). Patients with DRESS may require hospitalisation as the condition can be fatal. If signs

THIS DOCUMENT CONTAINS TRADE SECRETS, AND/OR COMMERCIAL INFORMATION, DELIVERED IN CONFIDENCE AND RELIANCE THAT SUCH INFORMATION WILL NOT BE COPIED OR MADE AVAILABLE TO ANY THIRD PARTY WITHOUT THE WRITTEN CONSENT OF SelectImmune PHARMA AB.

and symptoms of DRESS occur and no alternative aetiology can be identified, Kineret should be discontinued and other treatment considered.

8.3.4 Reference Document- Kineret®

The current version of the SmPC of July 2022 is the Reference Document and kept in the Investigator site file (ISF). In case of changes to the Reference Document, SelectImmune Pharma and the Coordinating Investigator has to re-evaluate the risk-benefit assessment.

8.3.5 Allowed concomitant medication

Interactions between Kineret® and other drugs have not been studied in formal studies. In clinical trials no interactions between Kineret® and other drugs have been observed, but Kineret® is not recommended in patients together with TNF-α-antagonists and Cytochrom P450-substrate.

8.4 Investigational Medicinal Product (IMP) - Nitrofurantoin Uro-Tablinen®

Product description

Marketed antibiotics investigational product will be used for the patients randomised to conventional antibiotics. The instructions in the product's SmPC will be followed such as storage, administration etc. Approved commercial products are used, which is why the labeling according to §5 (8) GCP-V is waived in the context of clinical trials.

Antibiotics used to treat uncomplicated cystitis.

-Genotoxicity

Nitrofurantoin is mutagenic in vitro in bacteria and in mammalian cells. The available in vivo data are contradictory, recent studies suggest at least weak mutagenicity in vivo. In long-term carcinogenicity studies in rats and mice, treatment-related tumorigenic effects in female mice (ovaries) and weak effects in male rats (testis and kidneys). The relevance of these findings to therapeutic use is not known. Since a mutagenic/carcinogenic potential cannot be ruled out at this time, Nitrofurantion should, if possible, not be used over a longer period of time and only if alternative therapies are not applicable.

-Carcinogenicity

Human data and extensive use of Nitrofurantoin over 50 years do not support carcinogicity of Nitrofurantoin Uro-Tablinen®. Suspicion of carcinogenicity cannot be excluded after long-term

THIS DOCUMENT CONTAINS TRADE SECRETS, AND/OR COMMERCIAL INFORMATION, DELIVERED IN CONFIDENCE AND RELIANCE THAT SUCH INFORMATION WILL NOT BE COPIED OR MADE AVAILABLE TO ANY THIRD PARTY WITHOUT THE WRITTEN CONSENT OF SelectImmune PHARMA AB.

application of Nitrofurantoin Uro-Tablinen. Thus, in this study the occurrence of carcinogenicity is unlikely.

-Fertility

At higher doses, nitrofurantoin impairs spermatogenesis. However, only female patients will be enrolled in this study.

-Embryo-fetal development

There are insufficient data to support the use of nitrofurantoin in pregnant women. Nitrofurantoin crosses the placental barrier. Animal studies have shown reproductive toxicity. The potential risk to humans is not known. The use of Nitrofurantoin Uro-Tablinen® is contraindicated during the last trimester of pregnancy (due to the risk of hemolytic anemia in the newborn). Nitrofurantoin Uro-Tablinen® must not be used during the first 6 months of pregnancy unless clearly required.

- Formulation Information

Oral retard pill

- Instructions for Use

Nitrofurantoin Uro-Tablinen® pills are taken orally during or after a meal with plenty of liquid.

8.4.1 Selection of dose

The dose of 100 mg Nitrofurantoin Uro-Tablinen® (retard effect, 50 mg) will be given according to the patients body weight (adapted to patient weight – daily dose 3,6 mg/kg patient weight) and will be then administered two times a day for 5 days.

8.4.2 Potential Toxicity in Patients

The following safety data of Nitrofurantoin Uro-Tablinen® refer to SmPC Nitrofurantoin Uro-Tablinen® 50 mg, Version June 2019.

<u>Tabulated summary of adverse reactions:</u>

The frequency of adverse reactions reflects treatment with Nitrofurantoin 30 mg administered 3 times a day according to EMA. Undesireble effects are stated in table below as rare (≥1/10,000 to <1/1,000).

THIS DOCUMENT CONTAINS TRADE SECRETS, AND/OR COMMERCIAL INFORMATION, DELIVERED IN CONFIDENCE AND RELIANCE THAT SUCH INFORMATION WILL NOT BE COPIED OR MADE AVAILABLE TO ANY THIRD PARTY WITHOUT THE WRITTEN CONSENT OF SelectImmune PHARMA AB.

Table 1: List of adverse drug reactions associated with Nitrofurantoin Uro-Tablinen®

**System Organ Class**	**Preferred term ordered by seriousness**	**Frequency**
Infections and parasitic diseases	Super Infections through Pseudomonas- or Candida species (on the urogenital tract limited)	Not known
Blood and the lymphatetic system	megaloblast Anemia	Not known
Blood and the lymphatetic system	Blood count changes (e.g. eosinophilia, thrombocytopenia, agranulocytosis and neutropenia, leucozytopenia, pancytopenia, acute hemolytic anemia)	< 1/10000
Immune system	Cutaneous vasculitis	Not known
Immune system	Autoimmune reactions (so-called lupus-like syndromes, "lupus-like syndromes", with symptoms like exanthema, arthralgia, eosinophilia, fever, positive Coombs-Test, antinuclear antibodies, antibodies against smooth muscle or against Glomeruli); anaphylactic/anaphylactoid reactions incl. anaphylactic shock, partly life-threatening	< 1/10000
Immune system	Allergic reactions: Drug fever	≥1/10
Psychiatric Diseases	Confusion, depression, euphoria and psychotic Reactions	≥ 1/10000
Nervous System	Increase of intracranial pressure	Not known
Nervous System	peripheral polyneuropathies (including optical neuritis) with partly irreversible symptoms muscle weakness, paresthesia and sensibility (hypesthesia, hypothermesthesia), drowsiness	< 1/10000

THIS DOCUMENT CONTAINS TRADE SECRETS, AND/OR COMMERCIAL INFORMATION, DELIVERED IN CONFIDENCE AND RELIANCE THAT SUCH INFORMATION WILL NOT BE COPIED OR MADE AVAILABLE TO ANY THIRD PARTY WITHOUT THE WRITTEN CONSENT OF SelectImmune PHARMA AB.

Nervous System	Headache (especially at the beginning of therapy) as well as dizziness, ataxia, nystagmus	≥1/10
Respiratory System	Pulmonary reactions: allergic pulmonary edema, pulmonary infiltration ("nitrofurantoin pneumonia"), interstitial pneumonia, pleurisy, dyspnea, cough, and chest pain.	≥1/100
Respiratory System	pulmonary fibrosis; asthma attacks	< 1/10000
Gastrointestinal disorders	Especially at the beginning of therapy: gastrointestinal complaints caused by central nervous system (loss of appetite, nausea, vomiting)	≥1/10
Gastrointestinal disorders	Diarrhoe (especially at the beginning of therapy)	≥ 1/10000
Gastrointestinal disorders	pancreatitis; parotitis	< 1/10000
Liver and Bile	Liver reactions: reversible cholestasis until life-threatening chronically active or granulomatous hepatitis, increase in transaminases	≥ 1/1000
Liver and Bile	liver necrosis with fatal liver failure, autoimmune Hepatitis	Not known
Skin and subcutaneous	allergic skin lesions (e.g., exanthema, pruritus, urticaria), angioedema	≥1/10
Skin and subcutaneous	Erythema nodosum, erythema multiforme, Stevens-Johnson-syndrome, Lyell's syndrome (sometimes life-threatening), transitory alopecia	< 1/10000
Kidney and urinary tract	Crystalluria	< 1/10000
Kidney and urinary tract	Interstitial Nephritis	Not known

THIS DOCUMENT CONTAINS TRADE SECRETS, AND/OR COMMERCIAL INFORMATION, DELIVERED IN CONFIDENCE AND RELIANCE THAT SUCH INFORMATION WILL NOT BE COPIED OR MADE AVAILABLE TO ANY THIRD PARTY WITHOUT THE WRITTEN CONSENT OF SelectImmune PHARMA AB.

Sexual organs and the mammary gland	reversible inhibition of spermatogenesis	< 1/10000
General diseases and complaints at the place of administration	Circulatory collapse	< 1/10000
General diseases and complaints at the place of administration	Harmless yellow or brown coloration of the urine	Not known

Please note that data on adverse reactions/adverse events may change over the time. For that reason, it is necessary that the investigator is familiar with the current version of the SmPC which is filed in the Investigator site file (ISF).

8.4.3 Authorization holder of study medication

The authorization holder of the study medication is Winthrop Arzneimittel GmbH. It will be provided by the local pharmacy (ATC code: J01XE01, Nitrofurantoin).

8.4.4 Drug Supply

The pharmacy of the clinic will provide sites with study medication on request by KKS. After the initiation visit, the center will be provided with study medication for patients.

Study medication must be received by designated personnel at the study site. The acknowledgement of receipt has to be sent back to KKS and pharmacy via fax and has to be stored in the ISF. The receipt of medication also has to be documented on the delivery log that is filed in the ISF.

8.4.5 Handling and storage of the medication

The medication has to be handled and stored safely and properly. It has to be kept in a secured location to which only the investigator and designated site personnel have access. The study medication has to be stored below 30°C. The min/max temperature has to be documented in the temperature log found in the ISF. The ready-to-use retard capsuels must remain in the outer carton in order to protect the contents from light.

8.4.6 Application of the study medicine

Study medication will be administered only by an authorized person at the investigator’s site.

THIS DOCUMENT CONTAINS TRADE SECRETS, AND/OR COMMERCIAL INFORMATION, DELIVERED IN CONFIDENCE AND RELIANCE THAT SUCH INFORMATION WILL NOT BE COPIED OR MADE AVAILABLE TO ANY THIRD PARTY WITHOUT THE WRITTEN CONSENT OF SelectImmune PHARMA AB.

The administration of study medication for each patient has to be documented on the drug accounting form found in the ISF. Destruction of study medication at site can take place only after sponsor approval.

8.4.7 Dose and adjustment of study treatment

The dose of 100 mg Nitrofurantoin Uro-Tablinen® (retard effect, 50 mg) will be each administered adapted to patient weight (daily dose 3,6 mg/kg patient weight) two times a day over 5 days.

8.4.8 Treatment after termination of study

The treatment after completion of the clinical trial (premature or regular) is decided individually between investigator and patient.

8.4.9 Management of toxicities of IMP

Toxicity: 100-120 mg to 2-year-olds did not produce any symptoms. 300 mg to 8 years of age gave moderate intoxication. Benign intracranial pressure increase in a 10-month-old child (75 mg daily for 1 week).

Symptoms: Nausea, vomiting, diarrhea. Headache, dizziness, ataxia, paresthesia, tremor. Pulmonary hypersensitivity reaction, exanthema, urticaria. Hemolytic anemia in predisposed persons. Polyneuropathy in people with renal impairment.

Treatment: If justified gastric emptying, carbon. Provide good diuresis. Alkaline urine speeds up the secretion. Symptomatic treatment. In case of polyneuropathy: treatment with vitamin B6. Liver and renal function should be monitored, as well as a complete blood count should be done.

8.4.10 Reference Document- Nitrofurantoin Uro-Tablinen®

The current version of the SmPC is kept in the Investigator site file (ISF). In case of changes to the Safety Reference document, SelectImmune Pharma and the coordinating investigator has to re-evaluate the risk-benefit assessment.

8.4.11 Allowed concomitant medication

1. Increased absorption with food or agents delaying gastric emptying.

2. Decreased absorption with magnesium trisilicate.

THIS DOCUMENT CONTAINS TRADE SECRETS, AND/OR COMMERCIAL INFORMATION, DELIVERED IN CONFIDENCE AND RELIANCE THAT SUCH INFORMATION WILL NOT BE COPIED OR MADE AVAILABLE TO ANY THIRD PARTY WITHOUT THE WRITTEN CONSENT OF SelectImmune PHARMA AB.

3. Decreased renal excretion of Nitrofurantoin by probenecid and sulfinpyrazone.

4. Decreased anti-bacterial activity by carbonic anhydrase inhibitors and urine alkalisation.

5. Anti-bacterial antagonism by quinolone anti-infectives.

6. Interference with some tests for glucose in urine.

7. As Nitrofurantoin belongs to the group of Antibacterials, it will have the following resulting interactions:

- Typhoid Vaccine (oral): Antibacterials inactivate oral typhoid vaccine.

8. In case of anaphylactic reactions: Treatment according to standard treatment guidelines: vasoactive substances (epinephrine, dopamine, norepinephrine, vasopressin), oxygen, volume administration, antihistaminkia (histamine H1 receptor antagonists), glucocorticoids

8.5 Drug Accountability and Compliance Checks

8.5.1 Drug Accountability Investigational Product

The investigator is fully responsible for the investigational products at the trial site, for maintaining adequate control of the investigational products, and for documenting all transactions with them.

An inventory (Individual Drug Accountability Form) must be kept tracking the investigational product given to each subject enrolled in the trial. This inventory log must be available during monitoring visits and will be checked by the monitor to ensure correct dispensing of the investigational product.

8.5.2 Trial Product Destruction

The Trial products will be destroyed by the pharmacy of the clinic.

9 Statistical Methods

9.1 Determination of Sample Size

One of the primary objective of the trial is to evaluate the safety and first signs of efficacy of Kineret. No formal sample size calculation evaluating the power of the trial has been performed. However, a consideration regarding the sample size was made as described below.

THIS DOCUMENT CONTAINS TRADE SECRETS, AND/OR COMMERCIAL INFORMATION, DELIVERED IN CONFIDENCE AND RELIANCE THAT SUCH INFORMATION WILL NOT BE COPIED OR MADE AVAILABLE TO ANY THIRD PARTY WITHOUT THE WRITTEN CONSENT OF SelectImmune PHARMA AB.

9.2 Definition of Trial Analysis Sets

All subjects enrolled in the trial (i.e. subjects for whom informed consent has been obtained) will be accounted for in the clinical trial report.

A full analysis set will comprise all enrolled subjects who meet the protocol-defined eligibility criteria.

A safety analysis set will be defined by excluding subjects from the full analysis set who either received no treatment with investigational product and/or for whom no post-baseline safety evaluations are available.

The decisions regarding inclusion/exclusion of subjects and/or subject data from the trial analysis sets will be documented in the clinical trial report.

9.3 Statistical Analysis

The statistical analysis wil be specified in a Statisitcal Analysis Plan prior to Database lock. All data analysis will be based on statistical hypothesis testing. Population variables, adverse events and efficacy variables will be formally evaluated. The primary objective of the trial is to evaluate the safety and efficacy of Anakinra treatment for episodes of acute cystitis in patients with recurrent disease. No formal sample size calculation evaluating the power of the trial has been performed. However, a consideration regarding the sample size was made based on previous studies of Anakinra in a murine acute cystitis model. For efficacy, the sample size was based on analysis of symptom scores, inflammatory parameters and bacterial cultures. A sample size of 20 patients in the Anakinra treatment group and ten in the group receiving antibiotics was deemed suitable to achieve criterion for significance (alpha) 0.05 and power 90% using the paired samples 1-tailed t-test. The null hypothesis is H0: mean change in symptom score = 0 and the alternative hypothesis is HA: mean change in symptom score > 0.

9.3.1 Clinical Laboratory Evaluation

For biochemistry and haematology parameters, the absolute value and the change from baseline to Visit 6 and baseline to Visit 7 will be summarised. In addition, the laboratory parameters will be classified as ‘low’, ‘normal’ or ‘high’, depending on whether the value is below, within or above the reference range, respectively. Shift tables will be produced showing the categories at baseline against those at Visit 6 and baseline to Visit 7.

THIS DOCUMENT CONTAINS TRADE SECRETS, AND/OR COMMERCIAL INFORMATION, DELIVERED IN CONFIDENCE AND RELIANCE THAT SUCH INFORMATION WILL NOT BE COPIED OR MADE AVAILABLE TO ANY THIRD PARTY WITHOUT THE WRITTEN CONSENT OF SelectImmune PHARMA AB.

9.3.2 General Principles

Non-parametric and parametric methods will be used according to the properties of the data set. All confidence intervals will be presented with 95% degree of confidence.

An observed cases approach will be used for tabulations of data by visit (i.e. involving only those subjects who attended each specific visit).

The end of treatment values will be presented for laboratory parameters (primary and secondary endpoints) and for efficacy data.

For tabulations on changes from baseline, baseline will be defined as the last assessment performed before application of Kineret.

Categorical data will be summarised using the number and percentage of subjects in each category. Continuous data will be summarised using the mean, 95% confidence interval for mean (CI), standard deviation (SD), median, minimum and maximum values.

Any changes from the statistical analysis planned in this clinical trial protocol will be described and justified in a protocol amendment and/or in the clinical trial report dependent on the type of deviation.

10 Data management

10.1 Data Protection

In this trial the “REGULATION (EU) 2016/679 OF THE EUROPEAN PARLIAMENT AND OF THE COUNCIL of 27 April 2016 on the protection of natural persons with regard to the processing of personal data and on the free movement of such data, and repealing Directive 95/46/EC (General Data Protection Regulation)” will be noted by all parties involved.

10.2 EDC-System (e-CRF)

The trial will use an electronic case report form (e-CRF/EDC-System) for data collection and documentation, which is hosted by KKS Marburg. The data are entered directly via web browser to the e-CRF and are transferred via encryption (HTTPS (TSL/SSL)) to the central database.

Access to the e-CRF is only allowed for persons who are documented as trial personnel. Each person who is allowed to make entries in the e-CRF receives a personal username and the URL for database login upon request (User-ID request). The initial password, which has to be

THIS DOCUMENT CONTAINS TRADE SECRETS, AND/OR COMMERCIAL INFORMATION, DELIVERED IN CONFIDENCE AND RELIANCE THAT SUCH INFORMATION WILL NOT BE COPIED OR MADE AVAILABLE TO ANY THIRD PARTY WITHOUT THE WRITTEN CONSENT OF SelectImmune PHARMA AB.

changed at first login, is transmitted automatically by email to the user upon request (Forgot Password?) to the personal email address, which is recorded in the system.

Before a user gets access to the productive environment, the user account is only activated for training. After the user has activated its account the user management at KKS enables the user for the appropriate site. The access level in the e-CRF depends on the group membership (investigator, study nurse, monitor, etc.). Thus, it is ensured that only authorized persons have access to the EDC system in order to document or monitor patient data. Users with monitoring function are not able to enter or change patient's data. They have the possibility to view the data write protected (review function) and they can use additional review functionality in case of any implausibility or questions/queries.

The completed e-CRF must be electronically signed (authorization) at the end of each visit by an investigator for each patient.

In addition, a final verification of a case form for each patient has to be performed by the principal investigator or the substitute. This final verification confirms that the patient's case report form is completely and accurately documented and reviewed by the investigator.

In order to ensure the anonymity of the patient data, the patient data in the e-CRF are recorded with a patient number consisting of a center number and a consecutive number. An allocation list (e.g. Rando-Log) containing the patient's patient number and the identifying data of the patient is only kept in the center.

Users of the EDC system receive training material (EDC Manual), which is provided by the KKS. The EDC-Manual is part of the ISF and contains detailed instructions for using the EDC system. If necessary KKS Marburg will provide additional training material and required documentation for the users. For training purpose of data entry and data review a training site is included in the database.

In a multistage procedure, the given data will be checked electronically for their plausibility and consistency. Even during data collection, implausible data will be flagged automatically by implemented validation checks. Detected inconsistencies and missing or implausible data will be clarified with queries (electronically) and necessary changes will be carried out.

The EDC system has an implemented audit trail. This assures that any documentation and/or changes to database items are traceable anytime.

The end of study is defined as Last Visit Last Subject (LVLS) and database closure. At the end of trial, the database will be closed after data cleaning process. This process will be documented according to SOPs of KKS Marburg.

The pseudonymized patient data recorded in the e-CRF are stored by KKS Marburg in accordance with legal requirements.

THIS DOCUMENT CONTAINS TRADE SECRETS, AND/OR COMMERCIAL INFORMATION, DELIVERED IN CONFIDENCE AND RELIANCE THAT SUCH INFORMATION WILL NOT BE COPIED OR MADE AVAILABLE TO ANY THIRD PARTY WITHOUT THE WRITTEN CONSENT OF SelectImmune PHARMA AB.

11 Administration

11.1 Source data and patient files

The investigator has to keep a written or electronic patient file for every patient participating in the clinical study. In this file, the available demographic and medical information of a patient has to be documented, in particular the following: name, year of birth, sex, height, weight, patient history, concomitant diseases and concomitant drug (including changes during the study), study identification, patient number, the date and process of informed consent, all study visit dates, predefined performed examinations and clinical findings, observed AEs and reason for withdrawal from the study if applicable. It should be possible to verify the inclusion and exclusion criteria for the study from the available data in this file and to identify each subject based on their patient file. All original printouts generated by technical equipment must also be archived as source data. All these documents must bear at least the patient identification and the print date printed out by the recording device in order to be able to assign them reliably. The printouts must be medically evaluated, dated and signed by the investigator.

For all data recorded, the source document must be defined in a source data agreement list at the trial site. There must only be one source defined at any time for any data elements. The list must be signed by the investigator and the CRA prior to trial start.

The trial monitor will check the eCRFs for accuracy and completeness by verifying data recorded in the CRF against source data to ensure such records are consistent.

Source data should, as a general rule be recorded in the subject's medical record or other defined document normally used at the trial site. Source data not normally collected as a routine part of the clinical practice at the site may be entered on a worksheet. Clinical assessments/safety evaluations must be signed by medically qualified (sub)investigators.

If the worksheet does not become part of the subject's medical record, the following should as a minimum be added to the subject's medical record:

For this clinical trial, the following parameters collected in the eCRF should be verifiable from source documents available at the trial site:

- Date of trial visits and date leaving the clinical trial
- Relevant medical history and diagnosis
- Nature of contraception used by the subject and result of pregnancy test(s), when applicable

THIS DOCUMENT CONTAINS TRADE SECRETS, AND/OR COMMERCIAL INFORMATION, DELIVERED IN CONFIDENCE AND RELIANCE THAT SUCH INFORMATION WILL NOT BE COPIED OR MADE AVAILABLE TO ANY THIRD PARTY WITHOUT THE WRITTEN CONSENT OF SelectImmune PHARMA AB.

- Data for evaluation of eligibility criteria
- Dispensation/administration of investigational product
- Non-investigational medicinal products used for each subject
- Concomitant medication (including changes) and diagnoses
- Subject demographics (sex, date of birth, race, ethnic origin)
- Clinical assessments (vital signs, physical examination, investigator’s assessments)
- Laboratory assessments (haematology, biochemistry and urine tests)
- Adverse events, (nature, dates)

In addition to the above, the following should be added to the subject’s medical record in chronological order:

- Date(s) of conducting the informed consent process including date of provision of subject information
- Subject screening number
- Subject randomisation number
- Investigational product dispensed
- The fact that the subject is participating in a clinical trial in patients with recurrent cystitis
- Other relevant medical information

11.2 Investigator Site File (ISF) and archiving

The investigator will be provided with an Investigator Site File (ISF) before start of the study by the KKS Marburg. This file contains all relevant documents necessary for the conduct of the study. This file with all study-related documents must be safely archived after termination of the study for at least 15 years or until SelectImmune pharma informs the investigator that the documents are no longer to be retained. It is the responsibility of the investigator to ensure that the patient identification list is stored for at least 15 years beyond the end of the clinical study. All original patient files must be stored for the longest possible time permitted by the regulations at the hospital.

The investigator must make arrangements to store essential trial documents, including the Investigator Site File.

In addition, the investigator is responsible for the archiving of all relevant source documents so that the trial data can be compared against source data after the completion of the trial (e.g. in case of an inspection from competent authorities).

THIS DOCUMENT CONTAINS TRADE SECRETS, AND/OR COMMERCIAL INFORMATION, DELIVERED IN CONFIDENCE AND RELIANCE THAT SUCH INFORMATION WILL NOT BE COPIED OR MADE AVAILABLE TO ANY THIRD PARTY WITHOUT THE WRITTEN CONSENT OF SelectImmune PHARMA AB.

The investigator is required to ensure the continued storage of the documents even if the investigator leaves the clinic/practice or retires before the end of the required storage period.

11.3 Monitoring, audit and inspection

The investigator will permit study-related monitoring and audits, Ethics Committee review, and regulatory inspections, providing direct access to source data/documents (see section 11.3.2).

11.3.1 Monitoring

The monitoring of the study takes place by the trained staff of the KKS. Patient recruitment cannot begin before the initiation visit. During the course of the study there will be onsite visits according to a prescheduled visit plan of the sponsor. The scope, frequency and depth of the monitoring will be specified in a study specific monitoring manual.

In general any discrepancies in the e-CRF should be discussed and clarified with the study team during the monitoring visit and corrections/additions should be done according to GCP requirements. Furthermore, at these visits problematic cases will be discussed.

Source data verification will be performed by direct access to the original patient records. The written informed consent to participate in this study includes the consent to a direct verification of the source data.

During the course of the trial, the monitor will visit the trial site to ensure that the protocol and GCP are adhered to, that all issues have been recorded to perform source data verification and to monitor drug accountability

The first monitoring visit should be performed as soon as possible after Visit 1 and no later than 2 weeks after.

The monitoring visit intervals will depend on the trial site's recruitment rate, the compliance of the trial site with the protocol and GCP.

In order to perform their role effectively, monitors and persons involved in quality assurance and inspections will need direct access to source data, e.g. medical records, laboratory reports, appointment books, etc. If the electronic medical record does not have a visible audit trail, the investigator must provide the monitor with signed and dated printouts. In addition, relevant site staff should be available for discussions at monitoring visits and between monitoring visits (e.g. by telephone).

THIS DOCUMENT CONTAINS TRADE SECRETS, AND/OR COMMERCIAL INFORMATION, DELIVERED IN CONFIDENCE AND RELIANCE THAT SUCH INFORMATION WILL NOT BE COPIED OR MADE AVAILABLE TO ANY THIRD PARTY WITHOUT THE WRITTEN CONSENT OF SelectImmune PHARMA AB.

11.3.2 Audit / Inspections

In compliance with European regulations/ICH-GCP Guidelines, it is required that the investigator and institution permit authorized representatives of the KKS, the Sponsor and the regulatory agency(ies) direct access to review any study-related documents and subject's original medical records for verification of study-related procedures and data during and/or after the study. The extent is permitted by the applicable laws and regulations and by signing a written informed consent, the subject is authorizing such access. Direct access includes examining, analyzing, verifying, and reproducing any records and reports that are important to the evaluation of the study. The investigator is responsible for giving any requested support for any inspection or audit visit. The Principal Investigator has to be available during these visits.

Audits and inspections may take place during or after the trial. The investigator and the site staff as well as KKS Giessen staff have an obligation to cooperate and assist in audits and inspections. If the trial site is contacted for an inspection by competent authorities, both KKS Giessen and SelectImmune Pharma must be notified immediately.

11.4 Protocol violation and discrepancies

Any protocol violation has to be recorded and documented either as “note” in the e-CRF or by using the study specific file notification form, which has to be signed and forwarded to the project manager. In the KKS all protocol violation will be tracked in a separate database. Prior to database soft lock, all protocol violation encountered throughout the study will be reviewed and their consequences on the efficacy evaluation will be assessed.

12 Handling of an Urgent Safety Measure

An Urgent Safety Measure is a measure taken to implement an action/protocol deviation under an emergency. This is defined within the EU Directive as *“…the occurrence of any new event relating to the conduct of the trial or the development of the investigational medicinal product where that new event is likely to affect the safety of the subjects, the sponsor and the investigator shall take appropriate urgent safety measures to protect the subjects against any immediate hazard.*” (Article 10(b) of Directive 2001/20/EC).

If the investigator becomes aware of information that necessitates an immediate change in the clinical trial procedure or a temporary halt to the clinical trial in order to protect clinical trial subjects from any immediate hazard to their health and safety, the investigator can do so without prior approval from SelectImmune Pharma, regulatory authority(ies) or IRB(s)/IEC(s).

THIS DOCUMENT CONTAINS TRADE SECRETS, AND/OR COMMERCIAL INFORMATION, DELIVERED IN CONFIDENCE AND RELIANCE THAT SUCH INFORMATION WILL NOT BE COPIED OR MADE AVAILABLE TO ANY THIRD PARTY WITHOUT THE WRITTEN CONSENT OF SelectImmune PHARMA AB.

The investigator must immediately inform KKS and SelectImmune Pharma - by contacting Professor Catharina Svanborg - of this change in the clinical trial procedure or of the temporary halt providing full details of the information and the decision-making process leading to the implementation of the urgent safety measure.

13 Completion of Trial

After completion of the trial, SelectImmune Pharma will prepare a clinical trial report in co-operation with the Coordinating Investigator and other Investigators as applicable. A summary of the study results will be uploaded into the EudraCT database.

13.1 Premature Termination of the Trial and/or Trial Site

SelectImmune Pharma, the investigator, the IRB/IECs or competent authorities may decide to stop the trial, part of the trial or a trial site at any time, but agreement on procedures to be followed must be obtained.Criteria and reporting procedures for premature termination is described in section 5.6.1 and 5.6.2.

13.2 Provision for Subject Care Following Trial Completion

After the completion of the trial, the subjects will be treated at the investigator’s discretion or referred to other physician(s) according to standard practice.

14 Ethical considerations

Good Clinical Practice (GCP) is an international ethical and scientific quality standard for designing, conducting, recording, and reporting studies that involve the participation of human patients. The study will be conducted in compliance with GCP and the applicable national regulations to assure that the rights, safety, and well-being of the participating patients are protected consistent with the ethical principles that have their origin in the Declaration of Helsinki.

15 Ethical and regulatory aspects

15.1 Ethics Committee (EC)

KKS certifies that written documentation of appropriate Ethics Committee approval of the protocol, patient information and informed consent will be obtained before the beginning of this study. This favorable opinion documents the date of the EC meeting, constitution of the committee and voting members present at the meeting as well as clearly identifies the trial, protocol version, and consent documents reviewed.

THIS DOCUMENT CONTAINS TRADE SECRETS, AND/OR COMMERCIAL INFORMATION, DELIVERED IN CONFIDENCE AND RELIANCE THAT SUCH INFORMATION WILL NOT BE COPIED OR MADE AVAILABLE TO ANY THIRD PARTY WITHOUT THE WRITTEN CONSENT OF SelectImmune PHARMA AB.

Any substantial amendments to the protocol and patient information will be submitted to the EC and they will be informed about SUSARs in accordance with national requirements. Additional trial sites may only recruit patients, if the KKS already obtained approval for the site. Where the clinical trial has been suspended or interrupted, the KKS informs the Ethics Committee, giving the reasons for suspension or interruption.

Within 90 days after termination of the clinical trial the KKS has to inform the leading EC (in case of suspending or interruption within 15 days).

The investigator must not begin the study until he/she has received written confirmation of approval by the EC.

15.2 Competent Authorities (CA)

15.2.1 Application to the national competent authority

Prior to commencement of the study, the study protocol will be submitted together with its associated documents to the Competent Authority for their approval. According to the German Drug Law (AMG) the study will only commence following provision of a written approval by the Competent Authority.

The KKS has to inform the competent authority before the beginning and within 90 days after termination of the clinical trial (in case of suspending or interruption within 15 days giving the reasons for suspension or interruption).

15.2.2 Notification to the local competent authority

According to §67 AMG the principal investigator and his deputy must inform the local authority about start and termination of the clinical trial.

Where the clinical trial has been suspended or interrupted by the KKS the principle investigator/deputy has to inform the local authority about this.

The principal investigator and deputy can delegate these responsibilities to the KKS. This must be documented and signed by each investigator.

15.3 Pre-conditions before study start

Before study start and recruitment of patients, following conditions have to be fulfilled:

- Favorable opinion to the study of the Ethics Committee (including investigators und deputy investigators) and of the Competent Authority

THIS DOCUMENT CONTAINS TRADE SECRETS, AND/OR COMMERCIAL INFORMATION, DELIVERED IN CONFIDENCE AND RELIANCE THAT SUCH INFORMATION WILL NOT BE COPIED OR MADE AVAILABLE TO ANY THIRD PARTY WITHOUT THE WRITTEN CONSENT OF SelectImmune PHARMA AB.

- Information to the Competent Authority (PEI) about the start of the study
- Notification to the local authorities about study start according to §67 AMG
- Signed study contract
- Initiation Visit
- Signed study protocol (by principal investigator, deputy principal investigator

15.4 Patient insurance

SelectImmune Pharma carries insurance covering participating study patients in the event of a trial-related injury except for claims arising from malpractive in accordance with applicable laws and regulations. Before the start of the clinical trial, an existing patient insurance policy must be proven. The patient insurance serves the financial protection of health damages of the study participants, which stand in direct connection with the clinical study. All relevant documentation regarding such insurance will be filed in the Trial Master File and at the site as appropriate.

THIS DOCUMENT CONTAINS TRADE SECRETS, AND/OR COMMERCIAL INFORMATION, DELIVERED IN CONFIDENCE AND RELIANCE THAT SUCH INFORMATION WILL NOT BE COPIED OR MADE AVAILABLE TO ANY THIRD PARTY WITHOUT THE WRITTEN CONSENT OF SelectImmune PHARMA AB.

Name and address of the insurer:	Chubb European Group SE<br>Lurgiallee 12<br>60439 Frankfurt am Main<br>Deutschland
Phone:	+49 (0) 69 756 130
Fax:	+49 (0) 69 764 193
Insurance Number:	DELSCA40639

In order not to jeopardize insurance coverage, the patient must immediately inform the insurance company or the investigator of any damage to health resulting from participation in the clinical trial. A copy of the complete insurance terms and conditions will be made available to the patient together with a copy of the signed patient information and consent form. The Sponsor shall maintain insurance coverage for the duration of the trial as required by applicable local regulations. An insurance certificate will be provided to the IEC.

All relevant documentation regarding such insurance will be filed in the Trial Master File and at each site as appropriate.

15.5 Investigator's responsibilities

The principal investigator at site has adequate experience in the conductance of clinical trials and designates a deputy with comparable qualification before the start of the study. He/she has to lead and supervise the trial site team (study team) set up for this study, which consists of qualified persons in the field of the study specific indication and GCP. The principal investigator is also obliged to forward any study specific information (e.g. study protocol, product information) or updates of these documents to the study team.

He/she is responsible for conducting the clinical study in accordance with the protocol, the ethical principles that have their origin in the Declaration of Helsinki (current version) as well as with the International Conference on Harmonization of Technical Requirements of Pharmaceuticals for Humans Use (ICH) Note for Guidance on Good Clinical Practice (ICH,

THIS DOCUMENT CONTAINS TRADE SECRETS, AND/OR COMMERCIAL INFORMATION, DELIVERED IN CONFIDENCE AND RELIANCE THAT SUCH INFORMATION WILL NOT BE COPIED OR MADE AVAILABLE TO ANY THIRD PARTY WITHOUT THE WRITTEN CONSENT OF SelectImmune PHARMA AB.

Topic E6 (R2), GCP-Guidelines, the relevant national laws and applicable regulatory requirements.

The principle investigator and the subinvestigators are responsible to conduct the designated duties in accordance with the protocol, especially concerning treatment of the patients, the AE/SAE announcement to KKS and related e-CRF documentation as well as according to the above mentioned regulations.

15.6 Further responsibilities

The coordinating investigator and the Sponsor may be contacted for medical questions and are responsible to choose qualified participating centers.

The KKS is the delegated representative of the sponsor and is responsible for project management, safety, monitoring and data management. The KKS is also responsible for all regulatory affairs matters such as ethics and authority approval and for the registration of the study and sets up the DSUR in cooperation with the sponsor, the statistician and the coordinating investigators. The main responsibility for the overall study remains with the sponsor.

Further responsibilities will be specified in separate contracts between the participating parties.

15.7 Patient information and consent

The consent of the patient to participate in the clinical study has to be given in writing before any study-related activities are carried out. It must be signed and personally dated by the patient after a reasonable decision phase and by the investigator designated by the principle investigator to conduct the informed consent discussion. A patient information sheet will be provided for the purpose of obtaining informed consent. It will be revised and forwarded to the patients whenever important new information becomes available that may be relevant to the consent of patients.

At the study center, the investigator will inform the patient verbally about the aim of the study, all study procedures, all risks and possible alternatives. The patients will also be informed of the data protection requirements. In doing so, the wording used will be chosen so that the information can be fully and readily understood by laypersons.

Provision of consent will be confirmed in the patient file by the investigator. The signed and dated declaration of informed consent will remain in the ISF and can be retrieved at any time

THIS DOCUMENT CONTAINS TRADE SECRETS, AND/OR COMMERCIAL INFORMATION, DELIVERED IN CONFIDENCE AND RELIANCE THAT SUCH INFORMATION WILL NOT BE COPIED OR MADE AVAILABLE TO ANY THIRD PARTY WITHOUT THE WRITTEN CONSENT OF SelectImmune PHARMA AB.

for monitoring, auditing and inspection purposes. A copy of the signed and dated information consent together with the insurance terms and conditions should be provided to the patient prior to participation. Only authorized physicians may inform the patients and obtain the consent for the trial.

Patients are informed that they are free to terminate the clinical trial at any time at their discretion without giving reasons and without prejudice.

In case of termination of treatment with study medication (but not for whole study), the subject will be informed verbally on the possibilities to continue study procedures (without further study medication). In general, the patient would receive best medical treatment. In case that the patient withdraws his consent for further treatment with study medication the patient will give her informed consent by means of a written, signed and dated re-consent form for the assessments. Only an investigator may obtain the consent for the assessment in accordance with the regulatory requirements and GCP principles. The completed re-consent form has to be filed in the ISF.

15.8 Changes to the study protocol

After the commencement of the clinical trial, the sponsor may make amendments to the protocol. If those amendments are substantial and are likely to have an impact on the safety of the trial subjects or to change the interpretation of the scientific documents in support of the conduct of the trial, or if they are otherwise significant, the sponsor shall notify the competent authorities and shall inform the ethics committee.[4]

Also the local authority must be informed by the investigator about any substantial amendments and changes of the principal investigator and the deputy principal investigator.

'If the opinion of the Ethics Committee is favorable and the competent authorities have raised no grounds for non-acceptance of the above mentioned substantial amendments, the sponsor shall proceed to conduct the clinical trial following the amended protocol.[5]

Changes of administrative or technical nature will be recorded in a document entitled “nonsubstantial amendment“. It will be sent for information to the relevant ECs and to the CA.

---

[4] DIRECTIVE 2001/20/EC OF THE EUROPEAN PARLIAMENT AND OF THE COUNCIL of 2 April 2001, Article 10 (a)

[5] DIRECTIVE 2001/20/EC OF THE EUROPEAN PARLIAMENT AND OF THE COUNCIL of 2 April 2001, Article 10 (a)

THIS DOCUMENT CONTAINS TRADE SECRETS, AND/OR COMMERCIAL INFORMATION, DELIVERED IN CONFIDENCE AND RELIANCE THAT SUCH INFORMATION WILL NOT BE COPIED OR MADE AVAILABLE TO ANY THIRD PARTY WITHOUT THE WRITTEN CONSENT OF SelectImmune PHARMA AB.

Amendments will be signed by all signatories of the protocol. All investigators will acknowledge the receipt and confirm by their signature on the amendment that they will adhere to the amendment. A copy of the signature page will be filed in the Trial Master File the original in the Investigator Site File.

15.9 Safety of subjects, immediate danger

In the light of the circumstances, notably the occurrence of any new event relating to the conduct of the trial or the development of the investigational medicinal product where that new event is likely to affect the safety of the subjects, the sponsor and the investigator shall take appropriate urgent safety measures to protect the subjects against any immediate hazard. The sponsor shall forthwith inform the competent authorities of those new events and the measures taken and shall ensure that the Ethics Committee is notified at the same time.[6]

Other administrative changes (not affecting the scope of the investigation or the scientific quality of the study) can be made following approval by the coordinating investigator; however the EC must be notified of these administrative protocol changes (as appropriate).

The principal investigator will be responsible for implementing any amendments at the study site (including the distribution of amendments to all staff concerned).

16 Financial aspects

The study is financed by SelectImmune Pharma.

17 Final report

The final report will be set up as a synopsis according to ICH E3 and will be submitted to the EC and CA within one year after termination of the study. The summary result will also be published in the EudrCT database. SelectImmune Pharma together with the Coordinating Investigator are responsible for the preparation of the report and KKS is responsible for submission to the EC and CA and to publish the result in the EudraCT database.

[6] DIRECTIVE 2001/20/EC OF THE EUROPEAN PARLIAMENT AND OF THE COUNCIL of 2 April 2001, Article 10(b)

THIS DOCUMENT CONTAINS TRADE SECRETS, AND/OR COMMERCIAL INFORMATION, DELIVERED IN CONFIDENCE AND RELIANCE THAT SUCH INFORMATION WILL NOT BE COPIED OR MADE AVAILABLE TO ANY THIRD PARTY WITHOUT THE WRITTEN CONSENT OF SelectImmune PHARMA AB.

18 Registration

Basic information of this clinical trial will be posted on the EudraCT public website before the first subject enters into the clinical trial. The KKS is responsible to inform the CA once the study is approved by the EC and then the study will be published in the database by the CA.

19 Publication

The results will be published in accordance with international guidelines and national law. In addition, the results will be presented at international congresses and published in international journal. Any publication of data will be prepared in collaboration between SelectImmune Pharma and the Coordinating Investigator and approved by both parties before publication. The time for each party to respond during this collaboration is agreed to a maximum of 4 weeks.

20 Use of Information

This clinical trial protocol as well as all other information, data and results relating to this clinical trial and/or to the investigational product(s) is confidential information belonging to SelectImmune Pharma and shall not be used by the investigator for purposes other than this clinical trial.

The investigator agrees that SelectImmune Pharma may use any and all information, data and results from this clinical trial in connection with the development of the investigational product(s) and, therefore, may disclose and/or transfer information, data and/or results to other investigators, regulatory authorities and/or commercial partners.

THIS DOCUMENT CONTAINS TRADE SECRETS, AND/OR COMMERCIAL INFORMATION, DELIVERED IN CONFIDENCE AND RELIANCE THAT SUCH INFORMATION WILL NOT BE COPIED OR MADE AVAILABLE TO ANY THIRD PARTY WITHOUT THE WRITTEN CONSENT OF SelectImmune PHARMA AB.

21 References

References

1. Ambite, I., et al., *Molecular Basis of Acute Cystitis Reveals Susceptibility Genes and Immunotherapeutic Targets.* PLoS Pathog, 2016. **12**(10): p. e1005848.
2. Cavalli, G. and C.A. Dinarello, *Anakinra Therapy for Non-cancer Inflammatory Diseases.* Front Pharmacol, 2018. **9**: p. 1157.
3. Fleischmann, R.M., et al., *Safety of extended treatment with anakinra in patients with rheumatoid arthritis.* Ann Rheum Dis, 2006. **65**(8): p. 1006-12.
4. Kullenberg, T., et al., *Long-term safety profile of anakinra in patients with severe cryopyrin-associated periodic syndromes.* Rheumatology (Oxford), 2016. **55**(8): p. 1499-506.
5. Singh, J.A., et al., *Risk of serious infection in biological treatment of patients with rheumatoid arthritis: a systematic review and meta-analysis.* Lancet, 2015. **386**(9990): p. 258-65.
6. Wawrysiuk, S., et al., *Prevention and treatment of uncomplicated lower urinary tract infections in the era of increasing antimicrobial resistance-non-antibiotic approaches: a systemic review.* Arch Gynecol Obstet, 2019. **300**(4): p. 821-828.
7. Vik, I., et al., *Ibuprofen versus pivmecillinam for uncomplicated urinary tract infection in women-A double-blind, randomized non-inferiority trial.* PLoS Med, 2018. **15**(5): p. e1002569.
8. Kronenberg, A., et al., *Symptomatic treatment of uncomplicated lower urinary tract infections in the ambulatory setting: randomised, double blind trial.* BMJ, 2017. **359**: p. j4784.
9. Gagyor, I., et al., *Ibuprofen versus fosfomycin for uncomplicated urinary tract infection in women: randomised controlled trial.* BMJ, 2015. **351**: p. h6544.
10. FDA. *Kineret*. 2001; Available from: https://www.accessdata.fda.gov/drugsatfda_docs/label/2001/anakamg111401LB.pdf.
11. Klunder, B., et al., *Population Pharmacokinetics of Upadacitinib Using the Immediate-Release and Extended-Release Formulations in Healthy Subjects and Subjects with Rheumatoid Arthritis: Analyses of Phase I-III Clinical Trials.* Clin Pharmacokinet, 2019. **58**(8): p. 1045-1058.
12. Bresnihan, B., et al., *Treatment of rheumatoid arthritis with recombinant human interleukin-1 receptor antagonist.* Arthritis Rheum, 1998. **41**(12): p. 2196-204.
13. Butler, D.S.C., et al., *Neuroepithelial control of mucosal inflammation in acute cystitis.* Sci Rep, 2018. **8**(1): p. 11015.

14. Alidjanov et al., ACSS-Fragebogen zur klinischen Diagnose und Verlaufsbeurteilung einer unkomplizierten Zystitis. Nieren- und Hochdruckkrankheiten, 2021 50(0): p. 1

THIS DOCUMENT CONTAINS TRADE SECRETS, AND/OR COMMERCIAL INFORMATION, DELIVERED IN CONFIDENCE AND RELIANCE THAT SUCH INFORMATION WILL NOT BE COPIED OR MADE AVAILABLE TO ANY THIRD PARTY WITHOUT THE WRITTEN CONSENT OF SelectImmune PHARMA AB.

22 List of Appendices

Appendix 1: Adverse Events Terminology

Appendix 2: Classification of Adverse Events

THIS DOCUMENT CONTAINS TRADE SECRETS, AND/OR COMMERCIAL INFORMATION, DELIVERED IN CONFIDENCE AND RELIANCE THAT SUCH INFORMATION WILL NOT BE COPIED OR MADE AVAILABLE TO ANY THIRD PARTY WITHOUT THE WRITTEN CONSENT OF SelectImmune PHARMA AB.

22.1 Appendix 1: Definitions and Terminology Associated with Clinical Safety Reporting in Clinical Trials

Adverse Event

An adverse event is defined as any untoward medical occurrence in a patient or clinical investigation subject administered a pharmaceutical product and which does not necessarily have a causal relationship with this treatment. An adverse event (AE) can therefore be any unfavourable and unintended sign (including an abnormal laboratory finding), symptom, or disease temporally associated with the use of a medicinal (investigational) product, whether or not related to the medicinal (investigational) product. (ICH Harmonized Tripartite Guideline for Good Clinical Practice, E6 (R2)).

This definition includes:

- accidental injuries, events related to trial procedures, reasons for any unfavourable and unplanned change in medication (drug and/or dose), clinically significant worsening of pre-existing conditions, or reasons for admission to hospital or surgical procedures unless these were planned before enrolment. It also includes AEs commonly observed and AEs anticipated based on the pharmacological effect of the investigational product. In addition, any laboratory abnormality assessed as clinically significant by the (sub)investigator must be recorded as an AE.

Adverse Drug Reaction (ADR) and Serious Adverse Drug Reaction (SADR)

An ADR is defined as all untoward and unintended response to an investigational medicinal product related to any dose administrated. For the definition of SADR refer to the definition of "Serious" below.

The phrase "responses to a medicinal products" means that a causal relationship between a medicinal product and an adverse event is at least a reasonable possibility, i.e., the relationship cannot be ruled out.

Serious Adverse Event (SAE)

An SAE is defined as any untoward medical occurrence that

- results in death.

THIS DOCUMENT CONTAINS TRADE SECRETS, AND/OR COMMERCIAL INFORMATION, DELIVERED IN CONFIDENCE AND RELIANCE THAT SUCH INFORMATION WILL NOT BE COPIED OR MADE AVAILABLE TO ANY THIRD PARTY WITHOUT THE WRITTEN CONSENT OF SelectImmune PHARMA AB.

- is life-threatening.
- requires inpatient hospitalisation or prolongation of existing hospitalisation. Planned hospitalisation or planned prolonged hospitalisation do not fulfil the criteria for being an SAE but should be documented in the subject's medical record.
- results in persistent or significant disability/incapacity.
- is a congenital anomaly/birth defect.

or

is a medically important condition. Events that may not be immediately life-threatening or result in death or hospitalisation but may jeopardise the subject or may require intervention to prevent one of the other outcomes listed in the definition above. Examples are intensive treatment in an emergency room or at home for allergic bronchospasm, blood dyscrasias and convulsions that do not result in hospitalization, development of drug dependency or drug abuse.

Unexpected Adverse Reaction (UAR)

An UAR is defined as an adverse reaction that is not consistent with the applicable product safety information (e.g. investigator's brochure for an unauthorised investigational product or summary of product characteristics for an authorised product).

Suspected Unexpected Serious Adverse Reactions (SUSAR)

A SUSAR is defined as a a suspected adverse drug reaction to an investigational medicinal product that is unexpected, serious and considered related.

Unexpected means not consistent with Investigator's Brochure or included in Summary of Product Characteristics.

THIS DOCUMENT CONTAINS TRADE SECRETS, AND/OR COMMERCIAL INFORMATION, DELIVERED IN CONFIDENCE AND RELIANCE THAT SUCH INFORMATION WILL NOT BE COPIED OR MADE AVAILABLE TO ANY THIRD PARTY WITHOUT THE WRITTEN CONSENT OF SelectImmune PHARMA AB.

22.2 Appendix 2: Classification of Adverse Events

Severity

The term severity is used to describe the intensity of a specific adverse event. The "Common Terminology Criteria for Adverse Events (CTCAE)", version 5.0, should be used to classify the severity of the AEs: [7]

Adverse events not listed in the CTCAE v5.0 should be graded as follows:

**Grade 1**	**Mild**, asymptomatic or mild symptoms; clinical or diagnostic observations only; intervention not indicated.
**Grade 2**	**Moderate,** minimal, local or noninvasive intervention indicated; limiting age-appropriate instrumental ADL*.
**Grade 3**	**Severe** or medically significant but not immediately life-threatening; hospitalization or prolongation of hospitalization indicated; disabling; limiting self care ADL**.
**Grade 4**	**Life-threatening** consequences; urgent intervention indicated.
**Grade 5**	**Death** related to AE.
Activities of Daily Living (ADL)<br>*Instrumental ADL refer to preparing meals, shopping for groceries or clothes, using the telephone, managing money, etc.<br>**Self care ADL refer to bathing, dressing and undressing, feeding self, using the toilet, taking medications, and not bedridden.	

Causality

It is the investigator's responsibility to determine whether there is a causal relationship between the adverse event and the administration of the study medication.

For causality assessment the **EU METHOD OF ASSESSMENT** should be used:[8]

---

[7] National Cancer Institute, 2017: Common Terminology Criteria for Adverse Events (CTCAE).Version 5.0. U.S. DEPARTMENT OF HEALTH AND HUMAN SERVICES, National Institutes of Health, National Cancer Institute. Internet publication: https://ctep.cancer.gov/protocolDevelopment/electronic_applications/docs/CTCAE_v5_Quick_Reference_8.5x11.pdf

[8] European Medicines Agency and Heads of Medicines Agencies, 2017: EU Individual Case Safety Report (ICSR) Implementation Guide. Section I.C.3.5.1.2, page 39.

THIS DOCUMENT CONTAINS TRADE SECRETS, AND/OR COMMERCIAL INFORMATION, DELIVERED IN CONFIDENCE AND RELIANCE THAT SUCH INFORMATION WILL NOT BE COPIED OR MADE AVAILABLE TO ANY THIRD PARTY WITHOUT THE WRITTEN CONSENT OF SelectImmune PHARMA AB.

CAUSALITY TERM (EU Method of Assessment)	EXAMPLE OF ASSESSMENT CRITERIA
**Reasonable possibility (Yes)**	• Event or laboratory test abnormality, with reasonable time relationship to drug intake.<br>• There is no reasonable explanation that the event is caused by disease or other drugs.
**No reasonable possibility (No)**	• Event or laboratory test abnormality, with a time to drug intake that makes a relationship improbable.<br>• Disease or other drugs provide plausible explanations.

Seriousness

The judgement as to whether the adverse event is serious is made by the reporting investigator.

Outcome

The outcome of adverse events has to be described by following criteria:[9]

- recovered/resolved
- recovering/resolving
- not recovered/not resolved
- recovered/resolved with sequelae
- fatal
- unknown

[9] Cf. European Medicines Agency, 2015: EMA/CHMP/ICH/287/1995. Committee for Human Medicinal Products. ICH guideline E2B (R3) on electronic transmission of individual case safety reports (ICSRs) - data elements and message specification - implementation guide. Step 5. Section E.i.7, page 87.

THIS DOCUMENT CONTAINS TRADE SECRETS, AND/OR COMMERCIAL INFORMATION, DELIVERED IN CONFIDENCE AND RELIANCE THAT SUCH INFORMATION WILL NOT BE COPIED OR MADE AVAILABLE TO ANY THIRD PARTY WITHOUT THE WRITTEN CONSENT OF SelectImmune PHARMA AB.

Each adverse event has to be followed up until: (whichever occurs first)

- the termination of the adverse event
- improving of the adverse event is not to be expected
- the database is closed

THIS DOCUMENT CONTAINS TRADE SECRETS, AND/OR COMMERCIAL INFORMATION, DELIVERED IN CONFIDENCE AND RELIANCE THAT SUCH INFORMATION WILL NOT BE COPIED OR MADE AVAILABLE TO ANY THIRD PARTY WITHOUT THE WRITTEN CONSENT OF SelectImmune PHARMA AB.

22.3 ACSS Questionnaire (German)

ACSS-Fragebogen

Erstvorstellung (Diagnose) – Teil A

Uhrzeit: ____UU:____MM Datum der Untersuchung: / / (Tag/Monat/Jahr)

		**Bitte geben Sie an, ob Sie unten genannte Symptome innerhalb der letzten 24 Stunden bemerkt haben, und bewerten Sie bitte deren Intensität** *(nur eine Antwort für jedes einzelne Symptom)*	**0**	**1**	**2**	**3**
**Typische Symptome**	**1**	Häufiges Wasserlassen mit geringen Urinportionen *(wiederholte WC-Besuche)*	☐ Nein<br>*bis 4-mal täglich*	☐ Ja, etwas öfter als sonst<br>*5-6-mal täglich*	☐ Ja, merklich öfter<br>*7-8-mal täglich*	☐ Ja, sehr oft<br>*9-10-mal täglich*
	**2**	Starker, unwillkürlicher Harndrang	☐ Nein	☐ Ja, wenig	☐ Ja, mäßig	☐ Ja, stark
	**3**	Schmerzen und Brennen beim Wasserlassen	☐ Nein	☐ Ja, wenig	☐ Ja, mäßig	☐ Ja, stark
	**4**	Gefühl einer unvollständigen Harnblasenentleerung	☐ Nein	☐ Ja, wenig	☐ Ja, mäßig	☐ Ja, stark
	**5**	Schmerzen oder Beschwerden *(unangenehmes Druckgefühl)* im Unterbauch oder Beckenbereich	☐ Nein	☐ Ja, wenig	☐ Ja, mäßig	☐ Ja, stark
	**6**	Sichtbares Blut im Urin	☐ Nein	☐ Ja, wenig	☐ Ja, mäßig	☐ Ja, stark
					**Gesamtpunktzahl =**	**Punkte**
**Differentialdiagnose**	**7**	Schmerzen in der Lendengegend *(Flanke)**	☐ Nein	☐ Ja, wenig	☐ Ja, mäßig	☐ Ja, stark
	**8**	Neuer oder zunehmender Ausfluss aus der Scheide	☐ Nein	☐ Ja, wenig	☐ Ja, mäßig	☐ Ja, stark
	**9**	Eitriger Ausfluss aus der Harnröhre *(unabhängig vom Wasserlassen)*	☐ Nein	☐ Ja, wenig	☐ Ja, mäßig	☐ Ja, stark
	**10**	Erhöhte Körpertemperatur *(über 37,5°C) /* Schüttelfrost	☐ Nein	☐ Ja, wenig	☐ Ja, mäßig	☐ Ja, stark
		Wenn Sie Temperatur gemessen haben, geben Sie diese bitte an	≤37,5 °C	37,6-37,9 °C	38,0-38,9 °C	≥39,0 °C
		* oft einseitig (auf einer Seite)			**Gesamtpunktzahl =**	**Punkte**

Lebensqualität

11 **Bitte geben Sie an, wie stark ausgeprägt die durch die oben genannten Symptome hervorgerufenen Beschwerden innerhalb der letzten 24 Stunden waren** *(wählen Sie bitte nur eine am ehesten zutreffende Antwort)***:**

☐ 0 Keine Beschwerden *(keine Symptome, fühle mich wie immer)*
☐ 1 Geringe Beschwerden *(fühle mich etwas unwohler als sonst)*
☐ 2 Starke Beschwerden *(fühle mich merklich schlechter als sonst)*
☐ 3 Sehr starke Beschwerden *(fühle mich schrecklich)*

12 **Bitte geben Sie an, wie weit die oben genannten Symptome Ihre alltägliche Aktivität / Leistungsfähigkeit innerhalb der letzten 24 Stunden beeinträchtigt haben** *(wählen Sie bitte nur eine am ehesten zutreffende Antwort)***:**

☐ 0 Überhaupt nicht beeinträchtigt *(arbeite wie an gewöhnlichen Tagen, ohne Beschwerden)*
☐ 1 Ein wenig beeinträchtigt *(wegen der Symptome arbeite ich etwas weniger)*
☐ 2 Bedeutend beeinträchtigt *(alltägliche Arbeit ist anstrengend geworden)*
☐ 3 Stark beeinträchtigt *(ich kann praktisch nicht arbeiten)*

13 **Bitte geben Sie an, wie weit die oben genannten Symptome Ihre gesellschaftlichen Aktivitäten (Besuche machen, sich mit Freunden treffen usw.) innerhalb der letzten 24 Stunden beeinträchtigt haben** *(wählen Sie bitte nur eine am ehesten zutreffende Antwort)***:**

☐ 0 Überhaupt nicht beeinträchtigt *(es hat sich nichts geändert, ich lebe so wie vorher)*
☐ 1 Ein wenig beeinträchtigt *(eine geringe Reduzierung der Aktivität)*
☐ 2 Bedeutend beeinträchtigt *(viel weniger aktiv, bleibe mehr zu Hause)*
☐ 3 Stark beeinträchtigt *(schrecklich, kann das Haus praktisch nicht verlassen)*

Gesamtpunktzahl = Punkte

Begleitumstände

**14**	**Bitte geben Sie an, ob zum Zeitpunkt des Ausfüllens des Fragenbogens bei Ihnen folgendes zutrifft:**		
	Menstruation *(Regel)* ?	☐ Nein	☐ Ja
	Prämenstruelle Beschwerden *(Beschwerden in der Zeit vor der Regel)* ?	☐ Nein	☐ Ja
	Klimakterisches Syndrom *(Beschwerden in den Wechseljahren)* ?	☐ Nein	☐ Ja
	Schwangerschaft?	☐ Nein	☐ Ja
	Zuckerkrankheit?	☐ Nein	☐ Ja

HALT

Bitte vergessen Sie nicht, den ausgefüllten Fragebogen Ihrem Arzt zurückzugeben.

Wir danken Ihnen für Ihre Mitarbeit!

Acute Cystitis Symptom Score (ACSS) © 2015. http://inter.interoco.com/copyright-depository/work-of-science/1013-1951954939.html

THIS DOCUMENT CONTAINS TRADE SECRETS, AND/OR COMMERCIAL INFORMATION, DELIVERED IN CONFIDENCE AND RELIANCE THAT SUCH INFORMATION WILL NOT BE COPIED OR MADE AVAILABLE TO ANY THIRD PARTY WITHOUT THE WRITTEN CONSENT OF SelectImmune PHARMA AB.

ACSS-Fragebogen

Kontrollvorstellung (Folgebefund) – Teil B

Uhrzeit: ____UU:____MM Datum der Untersuchung: / / (Tag/Monat/Jahr)

Bitte geben Sie an, ob Sie irgendwelche Änderungen in Ihrem Zustand seit dem Ausfüllen des ersten Teils unseres Fragebogens bemerkt haben? *(kreuzen Sie bitte Ihre Antwort an)*:

Dynamik

- ☐ 0 Ich fühle mich jetzt ausgezeichnet *(alle Symptome sind endgültig vergangen)*
- ☐ 1 Mir geht es jetzt wesentlich besser *(die Mehrheit der Symptome sind vergangen)*
- ☐ 2 Ich fühle mich jetzt nur gering besser *(die Mehrheit der Symptome sind immer noch da)*
- ☐ 3 Es gibt jetzt keine Änderung meines Zustands *(alle Symptome sind noch vorhanden)*
- ☐ 4 Es ist jetzt schlimmer geworden *(mein Zustand hat sich verschlechtert)*

Bitte geben Sie an, ob Sie unten genannte Symptome innerhalb der letzten 24 Stunden bemerkt haben, und bewerten Sie bitte deren Intensität *(nur eine Antwort für jedes einzelne Symptom)*:

			0	1	2	3
Typische Symptome	1	Häufiges Wasserlassen mit geringen Urinportionen *(wiederholte WC-Besuche)*	☐ Nein<br>*bis 4-mal täglich*	☐ Ja, etwas öfter als sonst<br>*5-6-mal täglich*	☐ Ja, merklich öfter<br>*7-8-mal täglich*	☐ Ja, sehr oft<br>*9-10-mal täglich*
	2	Starker, unwillkürlicher Harndrang	☐ Nein	☐ Ja, wenig	☐ Ja, mäßig	☐ Ja, stark
	3	Schmerzen und Brennen beim Wasserlassen	☐ Nein	☐ Ja, wenig	☐ Ja, mäßig	☐ Ja, stark
	4	Gefühl einer unvollständigen Harnblasenentleerung	☐ Nein	☐ Ja, wenig	☐ Ja, mäßig	☐ Ja, stark
	5	Schmerzen oder Beschwerden *(unangenehmes Druckgefühl)* im Unterbauch oder Beckenbereich	☐ Nein	☐ Ja, wenig	☐ Ja, mäßig	☐ Ja, stark
	6	Sichtbares Blut im Urin	☐ Nein	☐ Ja, wenig	☐ Ja, mäßig	☐ Ja, stark
					**Gesamtpunktzahl =**	**Punkte**
Differentialdiagnose	7	Schmerzen in der Lendengegend *(Flanke)**	☐ Nein	☐ Ja, wenig	☐ Ja, mäßig	☐ Ja, stark
	8	Neuer oder zunehmender Ausfluss aus der Scheide	☐ Nein	☐ Ja, wenig	☐ Ja, mäßig	☐ Ja, stark
	9	Eitriger Ausfluss aus der Harnröhre *(unabhängig vom Wasserlassen)*	☐ Nein	☐ Ja, wenig	☐ Ja, mäßig	☐ Ja, stark
	10	Erhöhte Körpertemperatur *(über 37,5°C)* / Schüttelfrost	☐ Nein	☐ Ja, wenig	☐ Ja, mäßig	☐ Ja, stark
		Wenn Sie Temperatur gemessen haben, geben Sie diese bitte an	≤37,5 °C	37,6-37,9 °C	38,0-38,9 °C	≥39,0 °C
		* oft einseitig (auf einer Seite)			**Gesamtpunktzahl =**	**Punkte**

Lebensqualität

11 **Bitte geben Sie an, wie stark ausgeprägt die durch die oben genannten Symptome hervorgerufenen Beschwerden innerhalb der letzten 24 Stunden waren** *(wählen Sie bitte nur eine am ehesten zutreffende Antwort)*:

- ☐ 0 Keine Beschwerden *(keine Symptome, fühle mich wie immer)*
- ☐ 1 Geringe Beschwerden *(fühle mich etwas unwohler als sonst)*
- ☐ 2 Starke Beschwerden *(fühle mich merklich schlechter als sonst)*
- ☐ 3 Sehr starke Beschwerden *(fühle mich schrecklich)*

12 **Bitte geben Sie an, wie weit die oben genannten Symptome Ihre alltägliche Aktivität / Leistungsfähigkeit innerhalb der letzten 24 Stunden beeinträchtigt haben** *(wählen Sie bitte nur eine am ehesten zutreffende Antwort)*:

- ☐ 0 Überhaupt nicht beeinträchtigt *(arbeite wie an gewöhnlichen Tagen, ohne Beschwerden)*
- ☐ 1 Ein wenig beeinträchtigt *(wegen der Symptome arbeite ich etwas weniger)*
- ☐ 2 Bedeutend beeinträchtigt *(alltägliche Arbeit ist anstrengend geworden)*
- ☐ 3 Stark beeinträchtigt *(ich kann praktisch nicht arbeiten)*

13 **Bitte geben Sie an, wie weit die oben genannten Symptome Ihre gesellschaftlichen Aktivitäten (Besuche machen, sich mit Freunden treffen usw.) innerhalb der letzten 24 Stunden beeinträchtigt haben** *(wählen Sie bitte nur eine am ehesten zutreffende Antwort)*:

- ☐ 0 Überhaupt nicht beeinträchtigt *(es hat sich nichts geändert, ich lebe so wie vorher)*
- ☐ 1 Ein wenig beeinträchtigt *(eine geringe Reduzierung der Aktivität)*
- ☐ 2 Bedeutend beeinträchtigt *(viel weniger aktiv, bleibe mehr zu Hause)*
- ☐ 3 Stark beeinträchtigt *(schrecklich, kann das Haus praktisch nicht verlassen)*

Gesamtpunktzahl = Punkte

Begleitumstände

14 **Bitte geben Sie an, ob zum Zeitpunkt des Ausfüllens des Fragenbogens bei Ihnen folgendes zutrifft:**

Menstruation *(Regel)*?	☐ Nein	☐ Ja
Prämenstruelle Beschwerden *(Beschwerden in der Zeit vor der Regel)*?	☐ Nein	☐ Ja
Klimakterisches Syndrom *(Beschwerden in den Wechseljahren)*?	☐ Nein	☐ Ja
Schwangerschaft?	☐ Nein	☐ Ja
Zuckerkrankheit?	☐ Nein	☐ Ja

HALT

Bitte vergessen Sie nicht, den ausgefüllten Fragebogen Ihrem Arzt zurückzugeben.

Wir danken Ihnen für Ihre Mitarbeit!

Acute Cystitis Symptom Score (ACSS) © 2015. http://inter.interoco.com/copyright-depository/work-of-science/1013-1951954939.html

THIS DOCUMENT CONTAINS TRADE SECRETS, AND/OR COMMERCIAL INFORMATION, DELIVERED IN CONFIDENCE AND RELIANCE THAT SUCH INFORMATION WILL NOT BE COPIED OR MADE AVAILABLE TO ANY THIRD PARTY WITHOUT THE WRITTEN CONSENT OF SelectImmune PHARMA AB.

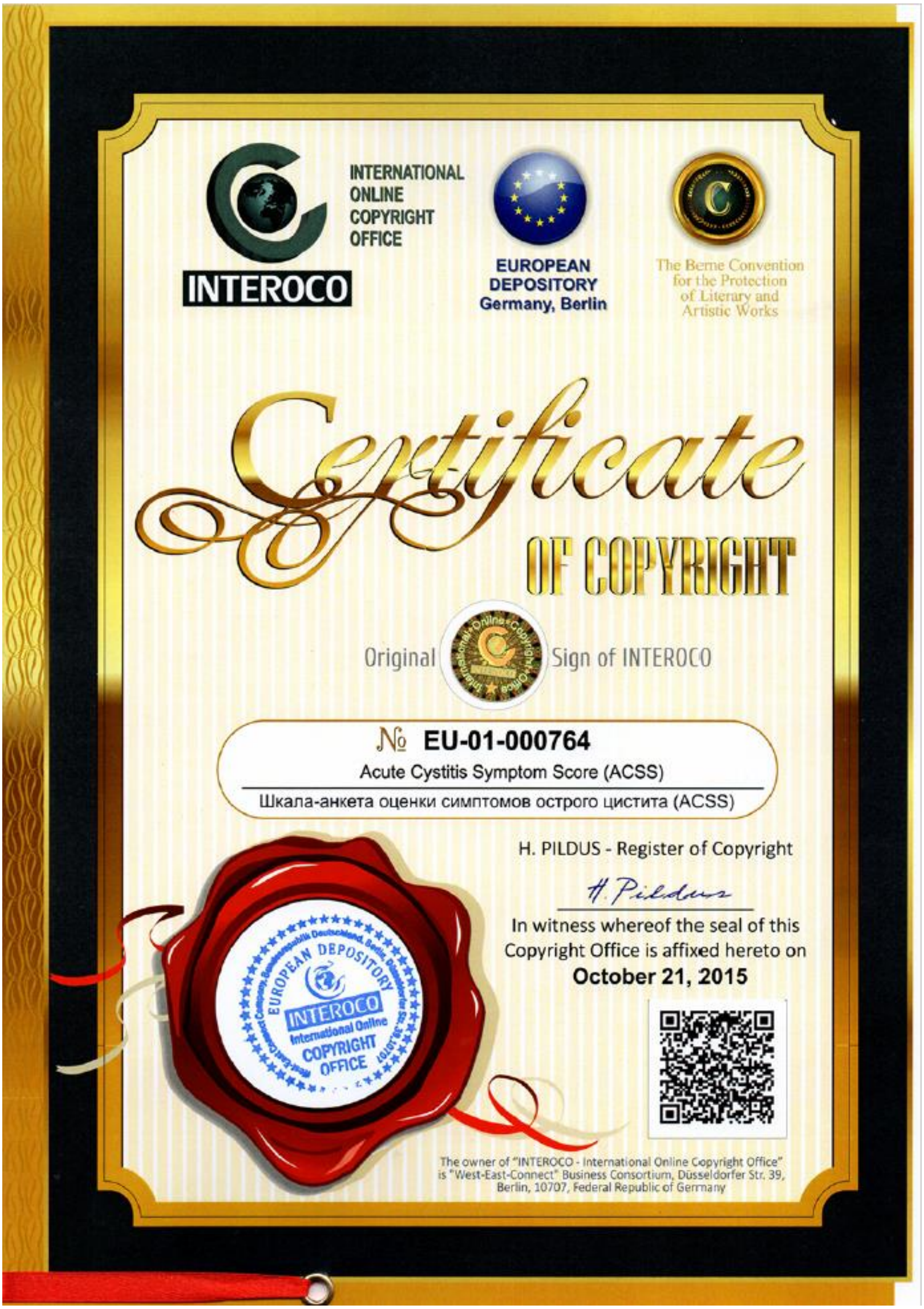
INTERNATIONAL ONLINE COPYRIGHT OFFICE

INTEROCO

EUROPEAN DEPOSITORY
Germany, Berlin

The Berne Convention for the Protection of Literary and Artistic Works

Certificate
OF COPYRIGHT

Original Sign of INTEROCO

№ EU-01-000764

Acute Cystitis Symptom Score (ACSS)

Шкала-анкета оценки симптомов острого цистита (ACSS)

H. PILDUS - Register of Copyright

H. Pildus

In witness whereof the seal of this Copyright Office is affixed hereto on
October 21, 2015

EUROPEAN DEPOSITORY
INTEROCO
International Online
COPYRIGHT
OFFICE

The owner of "INTEROCO - International Online Copyright Office" is "West-East-Connect" Business Consortium, Düsseldorfer Str. 39, Berlin, 10707, Federal Republic of Germany

THIS DOCUMENT CONTAINS TRADE SECRETS, AND/OR COMMERCIAL INFORMATION, DELIVERED IN CONFIDENCE AND RELIANCE THAT SUCH INFORMATION WILL NOT BE COPIED OR MADE AVAILABLE TO ANY THIRD PARTY WITHOUT THE WRITTEN CONSENT OF SelectImmune PHARMA AB.
